# Supplementary material for: Enhanced interpretation of immune cell phenotype and function through a rhesus macaque single-cell atlas
Source: Cell Genom. 2025 Apr 14;5(5):100849. doi: 10.1016/j.xgen.2025.100849 (PMC12143338; doi:10.1016/j.xgen.2025.100849)
Supplement: Document S1. Figures S1–S9 [file mmc1.pdf]

Cell Genomics, Volume *5*

Supplemental information

Enhanced interpretation of immune cell phenotype and function through a rhesus macaque single-cell atlas

Eisa Mahyari, Gregory J. Boggy, G.W. McElfresh, Maanasa Kaza, Sebastian Benjamin, Benjamin Varco-Merth, Sohita Ojha, Shana Feltham, William Goodwin, Candice Nkoy, Derick Duell, Andrea Selseth, Tyler Bennett, Aaron Barber-Axthelm, Jeremy V. Smedley, Caralyn S. Labriola, Michael K. Axthelm, R. Keith Reeves, Afam A. Okoye, Scott G. Hansen, Louis J. Picker, and Benjamin N. Bimber

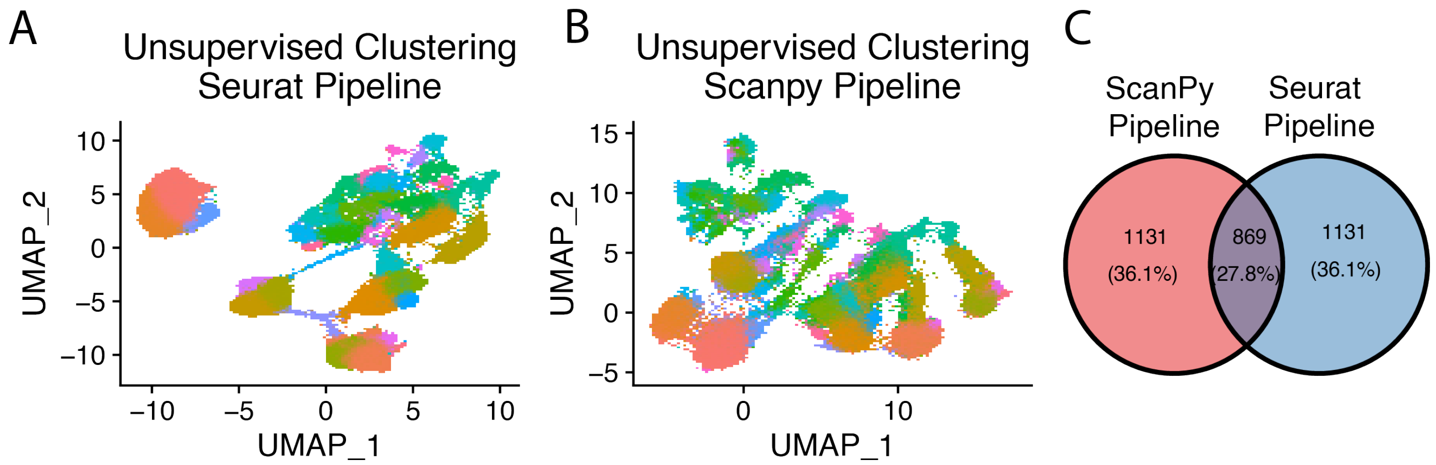

Figure S1. Comparison of dimensionality reduction between Seurat and ScanPy pipelines, related to Figure 5. The data used in these analyses are identical to Figure 5. Reference populations of peripheral T cells were sorted by FACS, producing 56,637 cells, followed by scRNA-seq. A) The data were processed using the pipeline implemented by the Seurat R package, with dimensionality reduction (PCA/UMAP) reference T cells, colored by population. Dimensionality reduction was performed on the top 3,000 variable genes. B) The same input data as (A), with processing and dimensionality reduction performed using the ScanPy python pipeline. Dimensionality reduction was performed on the top 3,000 variable genes. C) The Venn diagram shows the overlap between the top variable genes identified by the Seurat pipeline and the ScanPy pipeline. This difference will influence the outcome of PCA/UMAP and unsupervised clustering.

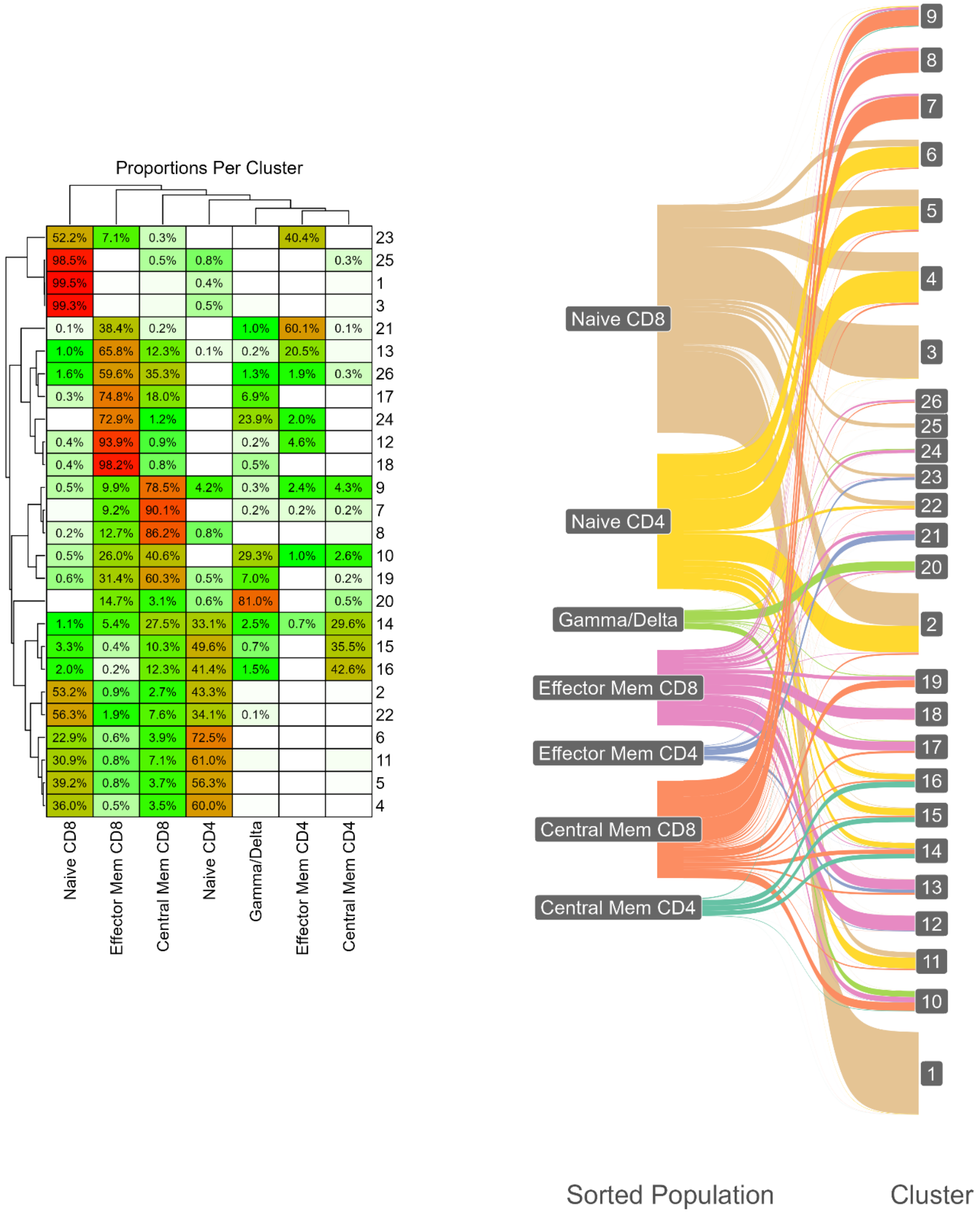

Figures S2. Comparison of unsupervised clustering using different scRNA-seq pipelines and algorithms, related to Figure 5. We performed unsupervised clustering on the data from Figure S1B (ScanPy pipeline) using the leiden clustering algorithm. The heatmap (left) and Sankey diagram (right) contrast the ground-truth population (defined by the cell sort) relative to the cluster assignment. While specific clusters can be generated that are pure or nearly pure for a single population, most clusters are a mixture of reference populations. These data demonstrate that the factors that drive the unsupervised clustering of T cells are not inherently aligned with established lineage definitions.

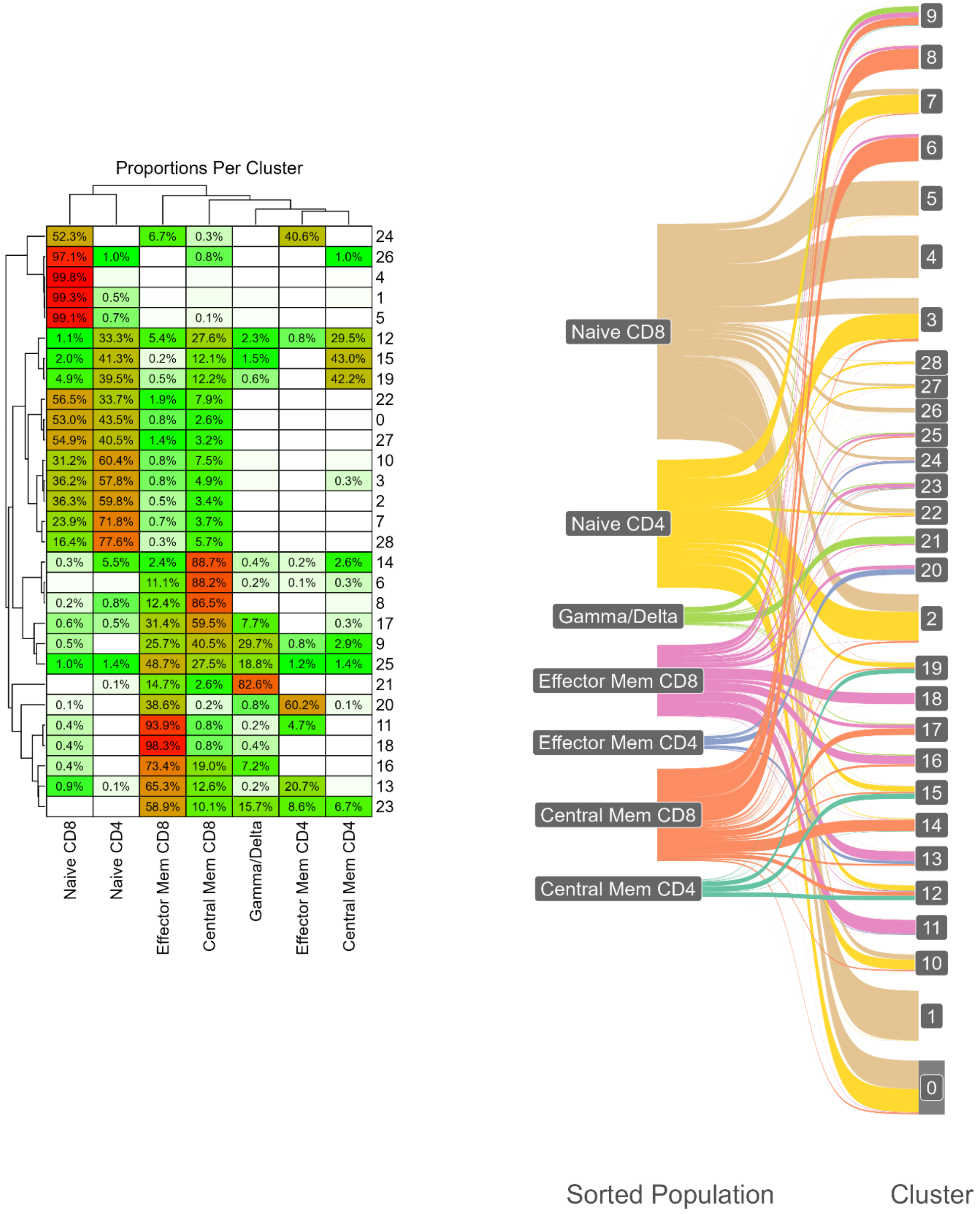

Figures S3. Comparison of unsupervised clustering using different scRNA-seq pipelines and algorithms, related to Figure 5. We performed unsupervised clustering on the data from Figure S1B (ScanPy pipeline) using the louvain clustering algorithm. The heatmap (left) and Sankey diagram (right) contrast the ground-truth population (defined by the cell sort) relative to the cluster assignment. While specific clusters can be generated that are pure or nearly pure for a single population, most clusters are a mixture of reference populations. These data demonstrate that the factors that drive the unsupervised clustering of T cells are not inherently aligned with established lineage definitions.

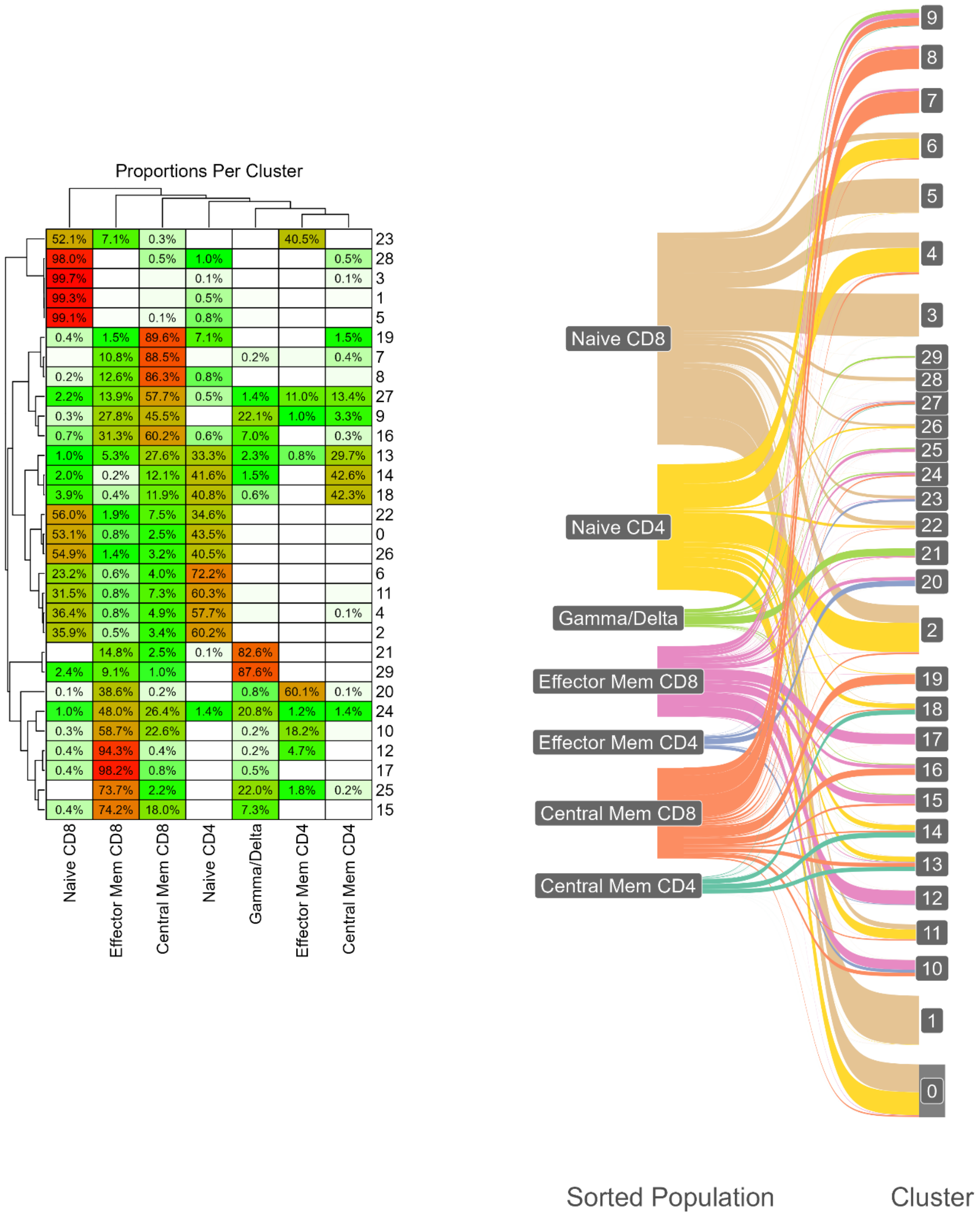

Figures S4. Comparison of unsupervised clustering using different scRNA-seq pipelines and algorithms, related to Figure 5. We performed unsupervised clustering on the data from Figure S1B (ScanPy pipeline) using the louvain-refined clustering algorithm. The heatmap (left) and Sankey diagram (right) contrast the ground-truth population (defined by the cell sort) relative to the cluster assignment. While specific clusters can be generated that are pure or nearly pure for a single population, most clusters are a mixture of reference populations. These data demonstrate that the factors that drive the unsupervised clustering of T cells are not inherently aligned with established lineage definitions.

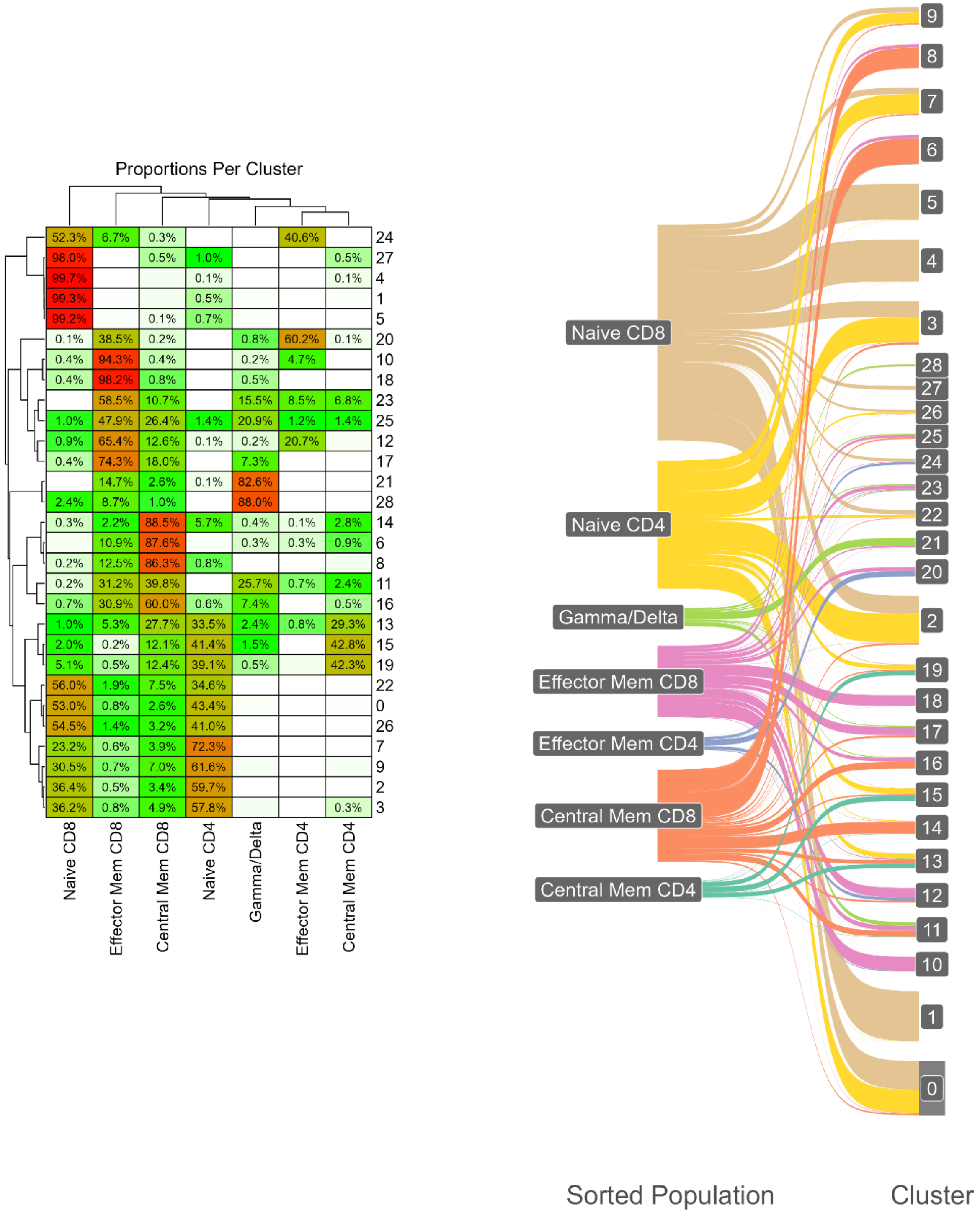

Figures S5. Comparison of unsupervised clustering using different scRNA-seq pipelines and algorithms, related to Figure 5. We performed unsupervised clustering on the data from Figure S1B (ScanPy pipeline) using the SLM clustering algorithm. The heatmap (left) and Sankey diagram (right) contrast the ground-truth population (defined by the cell sort) relative to the cluster assignment. While specific clusters can be generated that are pure or nearly pure for a single population, most clusters are a mixture of reference populations. These data demonstrate that the factors that drive the unsupervised clustering of T cells are not inherently aligned with established lineage definitions.

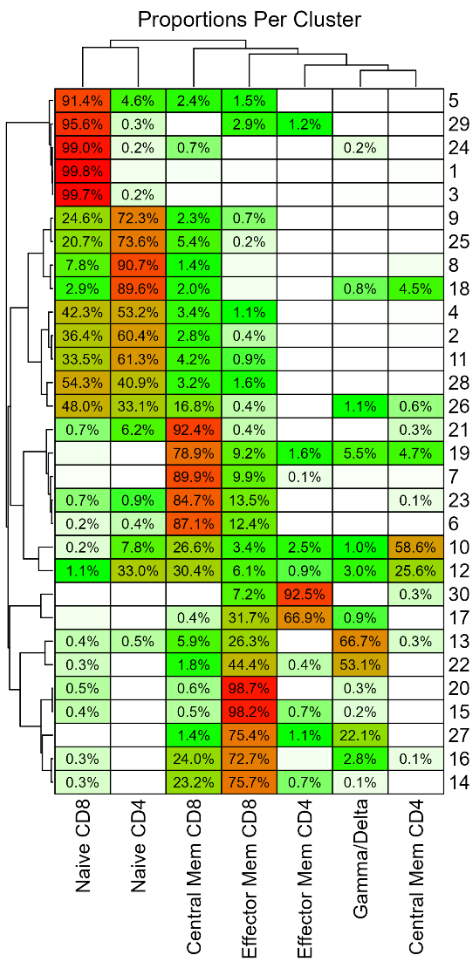

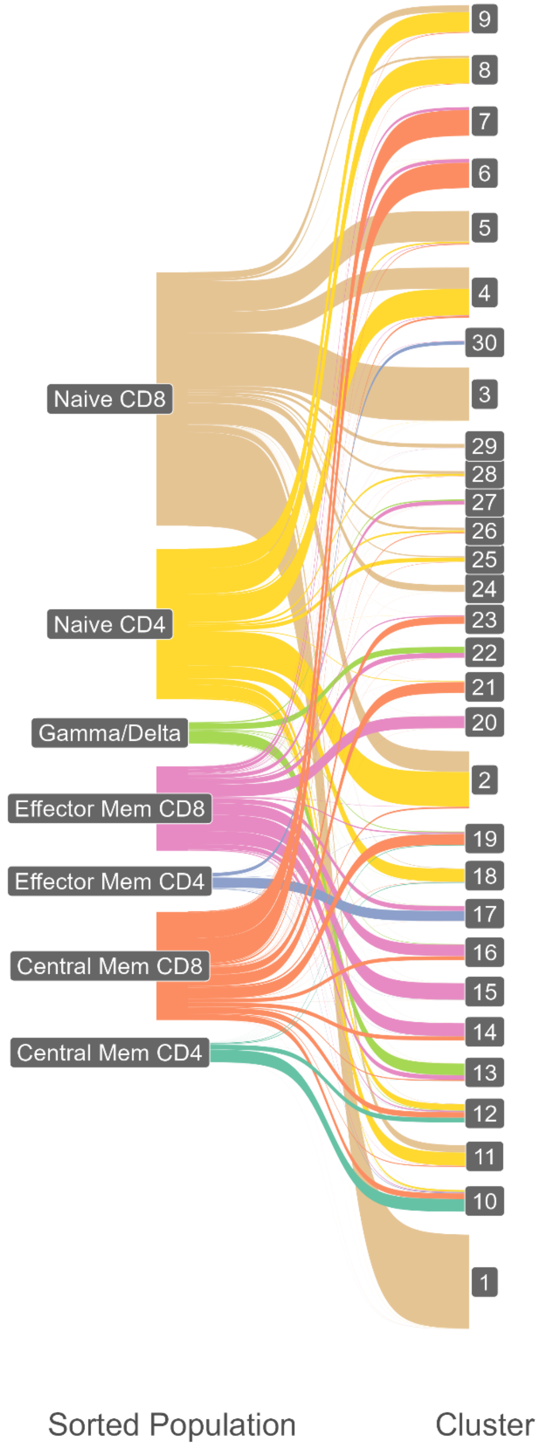

Figures S6. Comparison of unsupervised clustering using different scRNA-seq pipelines and algorithms, related to Figure 5. We performed unsupervised clustering on the data from Figure S1A (Seurat pipeline) using the leiden clustering algorithm. The heatmap (left) and Sankey diagram (right) contrast the ground-truth population (defined by the cell sort) relative to the cluster assignment. While specific clusters can be generated that are pure or nearly pure for a single population, most clusters are a mixture of reference populations. These data demonstrate that the factors that drive the unsupervised clustering of T cells are not inherently aligned with established lineage definitions.

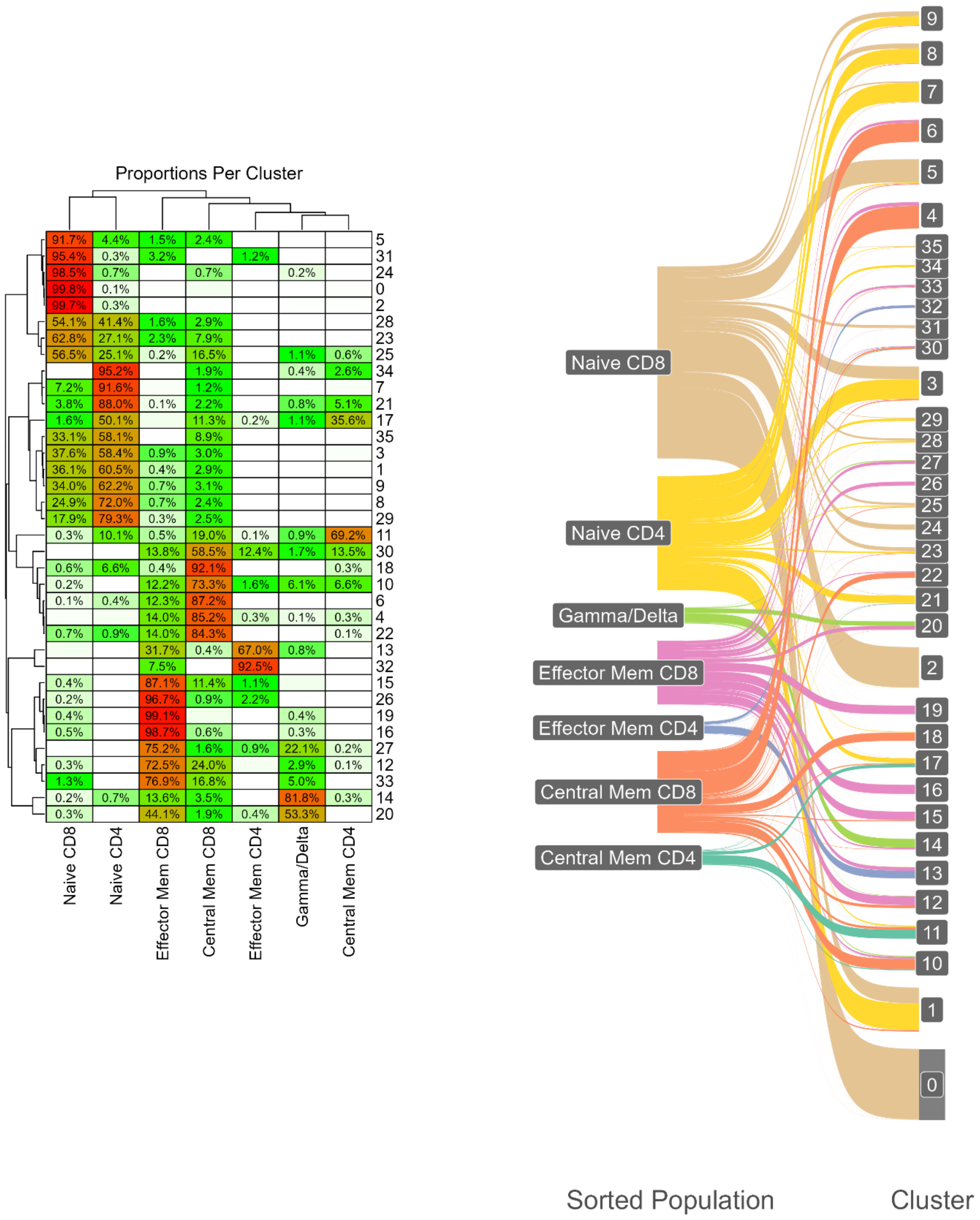

Figures S7. Comparison of unsupervised clustering using different scRNA-seq pipelines and algorithms, related to Figure 5. We performed unsupervised clustering on the data from Figure S1A (Seurat pipeline) using the louvain clustering algorithm. The heatmap (left) and Sankey diagram (right) contrast the ground-truth population (defined by the cell sort) relative to the cluster assignment. While specific clusters can be generated that are pure or nearly pure for a single population, most clusters are a mixture of reference populations. These data demonstrate that the factors that drive the unsupervised clustering of T cells are not inherently aligned with established lineage definitions.

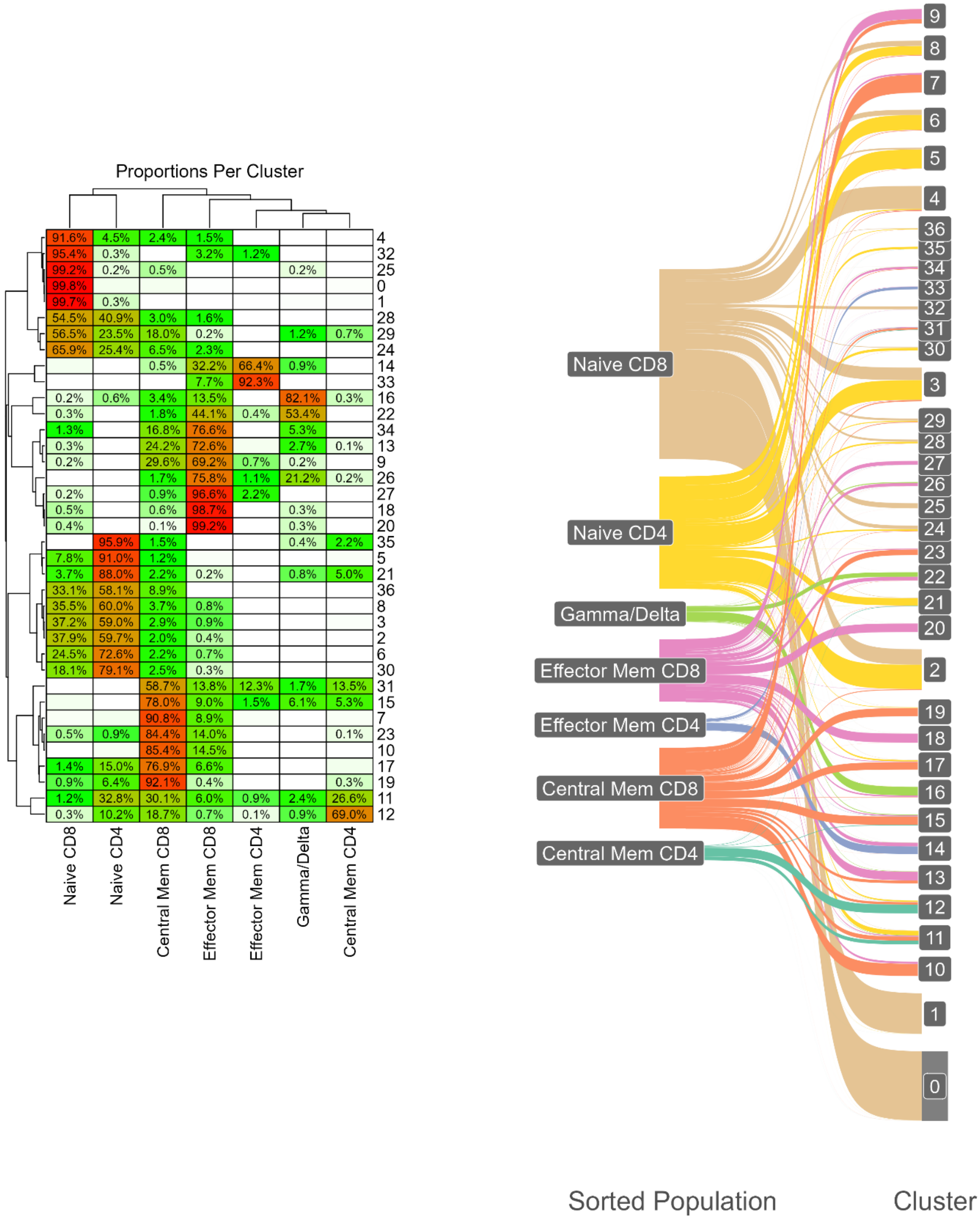

Figures S8. Comparison of unsupervised clustering using different scRNA-seq pipelines and algorithms, related to Figure 5. We performed unsupervised clustering on the data from Figure S1A (Seurat pipeline) using the louvain-refined clustering algorithm. The heatmap (left) and Sankey diagram (right) contrast the ground-truth population (defined by the cell sort) relative to the cluster assignment. While specific clusters can be generated that are pure or nearly pure for a single population, most clusters are a mixture of reference populations. These data demonstrate that the factors that drive the unsupervised clustering of T cells are not inherently aligned with established lineage definitions.

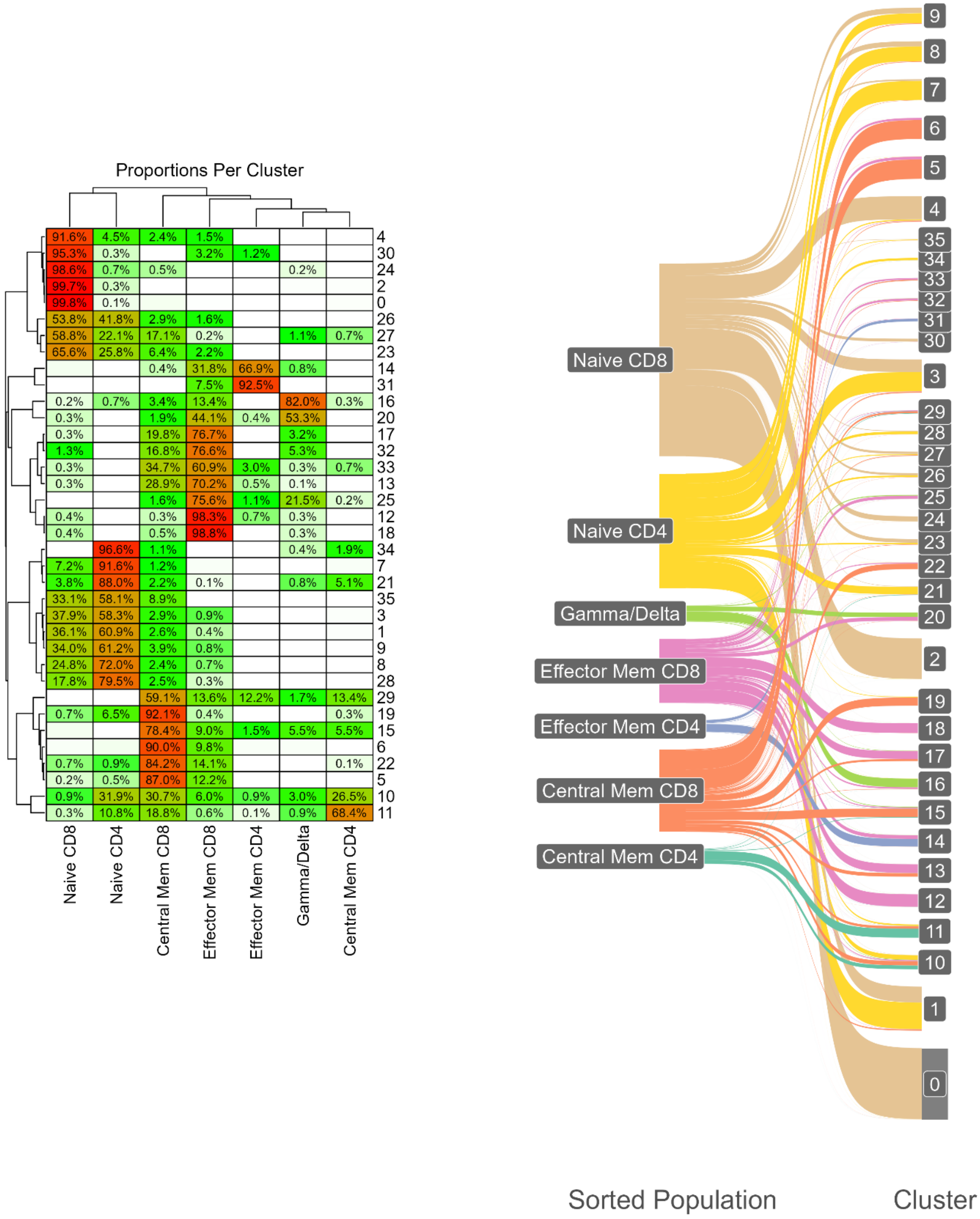

Figures S9. Comparison of unsupervised clustering using different scRNA-seq pipelines and algorithms, related to Figure 5. We performed unsupervised clustering on the data from Figure S1A (Seurat pipeline) using the SLM clustering algorithm. The heatmap (left) and Sankey diagram (right) contrast the ground-truth population (defined by the cell sort) relative to the cluster assignment. While specific clusters can be generated that are pure or nearly pure for a single population, most clusters are a mixture of reference populations. These data demonstrate that the factors that drive the unsupervised clustering of T cells are not inherently aligned with established lineage definitions.

Table S1. Table with demographics information for all rhesus macaques used in this study, related to Figure 1.

Table S2. List of CITE-seq antibodies and concentrations, related to STAR Methods.

Table S3. List of UCell gene modules used for cell type and state scoring, related to Figure 1.

Table S4. List of UCell gene modules used to detect mutually exclusive cell types, related to Figure 1.

Table S5. List of the genes and weights identified for the T cell Effector Differentiation Score (EDS), related to Figure 5.

Table S6. List of primers used to amplify macaque TCR alpha, beta, gamma, and delta sequences, related to STAR Methods.

Table S7. List of genes excluded from top variable genes prior to performing PCA, related to STAR Methods.

Table S8. The reference set of rhesus macaque V/D/J segments used for TCR detection, related to STAR Methods.

Table S9. List of all raw sequence datasets and NIH SRA accession numbers, related to STAR Methods.
