# Supplementary material for: Enhanced interpretation of immune cell phenotype and function through a rhesus macaque single-cell atlas
Source: Cell Genom. 2025 Apr 14;5(5):100849. doi: 10.1016/j.xgen.2025.100849 (PMC12143338; doi:10.1016/j.xgen.2025.100849)
Supplement: Note S1. Comparison of alternative scRNA-seq processing and clustering algorithms, related to Figure 5 [file mmc2.pdf]

Supplemental Note 1: Comparison of alternative scRNA-seq processing and clustering algorithms.

The choice of scRNA-seq pipeline, clustering algorithm(s) and parameters will impact the outcome of unsupervised analyses and the pattern of clustering[1]. To examine the impact of these choices in this study, and in particular to determine whether we should expect alternate data processing to create a pattern of clustering that more closely mirrors conventional immunologic populations, we processed the sorted reference T cell data (Figure 5), through multiple pipelines. We processed these data using both the Seurat R pipeline and the ScanPy python pipeline, followed by unsupervised clustering using multiple approaches (Figure S1)[2,3]. Not surprisingly, the two pipelines produced slightly different patterns of clustering (Figure S1A-B). It is worth noting that one difference in the Seurat and ScanPy pipelines is the algorithm used to identify the top variable genes in a dataset (Figure S1C). In dominant scRNA-seq pipelines, the top variable genes are passed to PCA and therefore changes in this list will influence downstream analyses.

As we demonstrated in the main text, the result of unsupervised clustering does not inherently align with established immune subsets. To examine whether changes to the clustering algorithm or parameters could impact this, we performed unsupervised clustering on both the Seurat and ScanPy data using four different algorithms: leiden, louvain, louvain refined, and SLM. The results of these are shown in Figures S2-S9, where each figure shows the results from one pipeline and algorithm. For each iteration, we contrasted the resulting clusters against reference population as a source of ground truth. As can be seen from the heatmaps, while it is possible for clusters to exist that contain a single reference population, is it more common for a cluster to be a mixture of multiple populations. Consistent with the main text, naïve CD4 and CD8 T cells were more likely to cluster independently than memory subsets. The Sankey plots illustrate the same point. These analyses continue to demonstrate that the transcriptomic features that govern neighborhood clustering do not inherently align with canonical definitions of immune cell subsets.

LITERATURE CITED

1. Rich, J.M., Moses, L., Einarsson, P.H., Jackson, K., Luebbert, L., Booeshaghi, A.S., Antonsson, S., Sullivan, D.K., Bray, N., Melsted, P., and Pachter, L. (2024). The impact of package selection and versioning on single-cell RNA-seq analysis. bioRxiv. 10.1101/2024.04.04.588111.

2. Butler, A., Hoffman, P., Smibert, P., Papalexi, E., and Satija, R. (2018). Integrating single-cell transcriptomic data across different conditions, technologies, and species. Nat Biotechnol *36*, 411-420. 10.1038/nbt.4096.
3. Wolf, F.A., Angerer, P., and Theis, F.J. (2018). SCANPY: large-scale single-cell gene expression data analysis. Genome Biol *19*, 15. 10.1186/s13059-017-1382-0.
