# Supplementary material for: Enhanced interpretation of immune cell phenotype and function through a rhesus macaque single-cell atlas
Source: Cell Genom. 2025 Apr 14;5(5):100849. doi: 10.1016/j.xgen.2025.100849 (PMC12143338; doi:10.1016/j.xgen.2025.100849)
Supplement: Document S2. Article plus supplemental information [file mmc12.pdf]

Cell Genomics

Resource

Enhanced interpretation of immune cell phenotype and function through a rhesus macaque single-cell atlas

Graphical abstract

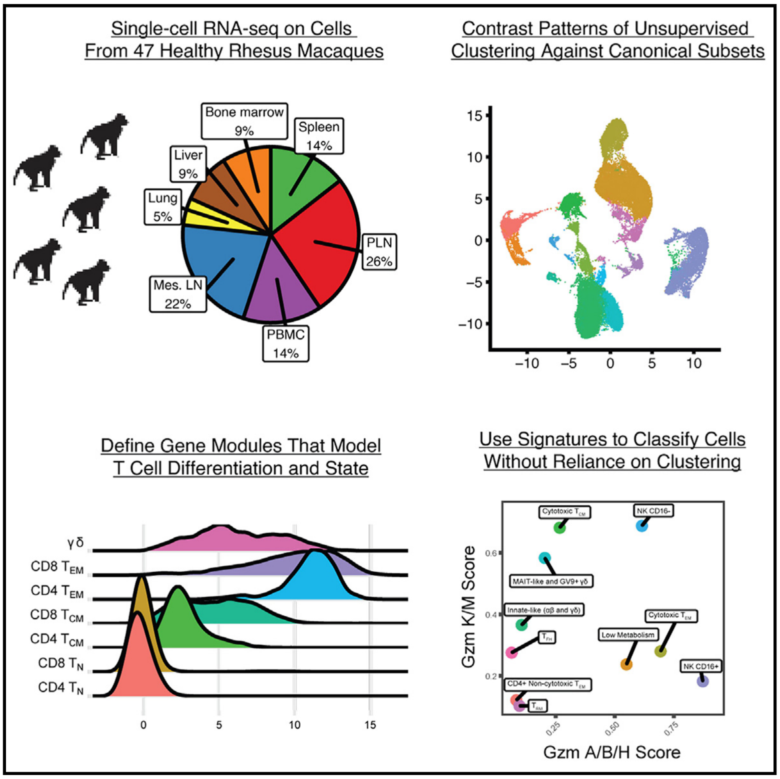

Authors

Eisa Mahyari, Gregory J. Boggy, G.W. McElfresh, ..., Scott G. Hansen, Louis J. Picker, Benjamin N. Bimber

Correspondence

bimber@ohsu.edu

In brief

Mahyari et al. generated a comprehensive immune cell single-cell RNA-sequencing (scRNA-seq) atlas for rhesus macaques. They contrasted patterns of unsupervised clustering against canonical functional and lineage subsets, highlighting areas of systematic difference between scRNA-seq and other technologies. These findings highlight important considerations for the interpretation of scRNA-seq in any species.

Highlights

- Generated rhesus macaque single-cell RNA-seq immune atlas
- Defined major immune subsets and RNA-centric markers
- Contrasted transcriptomic profiles against established surface marker definitions
- Validated gene modules and components that model T cell differentiation and state

Mahyari et al., 2025, Cell Genomics 5, 100849
May 14, 2025 © 2025 The Author(s). Published by Elsevier Inc.
https://doi.org/10.1016/j.xgen.2025.100849

CellPress

Cell Genomics

CellPress
OPEN ACCESS

Resource

Enhanced interpretation of immune cell phenotype and function through a rhesus macaque single-cell atlas

Eisa Mahyari,[1] Gregory J. Boggy,[1] G.W. McElfresh,[1] Maanasa Kaza,[1] Sebastian Benjamin,[1] Benjamin Varco-Merth,[1,2] Sohita Ojha,[2] Shana Feltham,[2] William Goodwin,[1,2] Candice Nkoy,[1,2] Derick Duell,[1,2] Andrea Selseth,[2] Tyler Bennett,[2] Aaron Barber-Axthelm,[2] Jeremy V. Smedley,[1,2] Caralyn S. Labriola,[1,2] Michael K. Axthelm,[1,2] R. Keith Reeves,[3,4] Afam A. Okoye,[1,2] Scott G. Hansen,[1,2] Louis J. Picker,[1,2] and Benjamin N. Bimber[1,2,5,*]
[1]Oregon National Primate Research Center, Oregon Health and Science University, Beaverton, OR 97006, USA
[2]Vaccine and Gene Therapy Institute, Oregon Health and Science University, Beaverton, OR 97006, USA
[3]Division of Innate and Comparative Immunology, Center for Human Systems Immunology, Duke University School of Medicine, Durham, NC, USA
[4]Department of Surgery, Duke University School of Medicine, Durham, NC, USA
[5]Lead contact
*Correspondence: bimber@ohsu.edu
https://doi.org/10.1016/j.xgen.2025.100849

SUMMARY

Single-cell RNA sequencing (scRNA-seq) allows cell classification using genome-wide transcriptional state; however, high-dimensional transcriptomic profiles, and the unsupervised analyses employed to interpret them, provide a systematically different view of biology than well-established functional/lineage definitions of immunocytes. Understanding these differences and limits is essential for accurate interpretation of these rich data. We present the Rhesus Immune Reference Atlas (RIRA), the first immune-focused macaque single-cell multi-tissue atlas. We contrasted transcriptional profiles against immune lineages, using surface protein and marker genes as ground truth. While the pattern of clustering can align with cell type, this is not always true. Especially within T and natural killer (NK) cells, many functionally distinct subsets lack defining markers, and strong shared expression programs, such as cytotoxicity, result in systematic intermingling by unsupervised clustering. We identified gene programs with high discriminatory/diagnostic value, including multi-gene signatures that model T/NK cell maturation. Directly measuring these diagnostic programs complements unsupervised analyses.

INTRODUCTION

Cells of the immune system have been the subject of intense study using a range of experimental techniques. There is a wealth of knowledge about immune cell lineage differentiation and functional properties.[1] For decades, flow cytometry has been the dominant platform for the study of immune cells, typically measuring a small number of protein markers (commonly less than 14, although higher-plex systems can be used).[2] While this is vastly smaller than the features measured by transcriptome-wide techniques, markers are generally selected to include proteins that either directly mediate or are correlated with a relevant biological property, including proteins that define fundamental immune subsets/lineages (e.g., CD3, CD14, CD16, CD20), proteins involved in specific modes of antigenic recognition (e.g., major histocompatibility complex [MHC] type, T cell receptor [TCR], immunoglobulin), co-stimulation (e.g., CD27, CD28), migratory capacity or tissue localization (e.g., CCR5, CCR7, CXCR5, CD62L, $\alpha e\beta 7$), activation or proliferation (e.g., CD71, Ki-67), cell survival (e.g., CD95), or function (e.g., CD40L, cytokines, specific restriction factors). Thus, while the number of markers per experiment is relatively low, their ability to characterize functionally important immune cell properties is significant. Further, definitions based on marker genes are straightforward to describe in literature and easy to reproduce. Even if there are clear limits to definitions based on a handful of markers, this transferability is critical for the development of a common immunologic understanding.

Single-cell RNA sequencing (scRNA-seq), which measures thousands of genes per cell, has obvious benefits in terms of characterizing the functional and differentiation state of cells. Nonetheless, there are systematic differences between scRNA-seq profiles and well-established marker-gene-based definitions of immune cells. These differences, which result from technical, biological, and bioinformatic causes, must be understood to accurately interpret scRNA-seq data and put these data into the context of existing immunologic knowledge. Differential regulation between RNA and protein within the cell can create discordance between surface protein profiles and scRNA-seq data.[3,4] For example, key surface markers that are

 © 2025 The Author(s). Published by Elsevier Inc.
This is an open access article under the CC BY license (http://creativecommons.org/licenses/by/4.0/).

robust at the protein level, such as CD4, are less reliable when measured by RNA.[5] A more subtle difference is that antibody- or probe-based reagents specifically bind their target, meaning the relative abundance of the protein is less critical. In contrast, the random sampling of RNA molecules in most scRNA-seq methods means that mRNA of genes with higher cellular expression will be detected more consistently.

The dominant modes of data analysis create a second layer of difference. Analysis of scRNA-seq data commonly employs unsupervised techniques developed for high-dimensional data, such as principal-component analysis (PCA)/uniform manifold approximation and projection (UMAP) or non-negative matrix factorization.[6–8] These methods are extremely powerful for the identification of sources of transcriptional diversity within data, grouping transcriptionally similar cells, and they provide data-driven discovery tools. Nonetheless, the biological processes that drive the strongest variance in the transcriptome, which is what dominant scRNA-seq analysis pipelines are designed to detect, are not inherently aligned with the transcriptional programs that define cell lineages or function, and distinct cellular subsets often share gene expression programs. As a result, unsupervised clustering can intermingle cell type and cell state. Thus, the assumption that cells with transcriptional profiles that are closely related in PCA space are functionally similar is not always true. While highly dissimilar cell types tend to have clearly distinct transcriptomes (e.g., B cells, T cells, and monocytes), and are therefore reliably separated, we will show this is less consistent at finer resolutions. In addition to the challenges associated with interpreting scRNA-seq profiles in the context of established cellular phenotypes, there are challenges associated with reproducibility and interoperability. While unsupervised analyses are a powerful tool, the pattern of clustering is dependent on the input data. While this “discovery” of dataset-specific signals is arguably the point, the lack of a stable, easily transferable definition of cell phenotype limits our ability to measure the identical phenotype between experiments or across laboratories.

Reference datasets can, in theory, help these challenges by providing curated transcriptional profiles for key cell types. Algorithms exist to align experimental data against a well-annotated reference dataset and transfer cell type labels from the literature.[9,10] Nonetheless, it is common for a single-cell reference atlas to merge cells from a wide range of tissues, perform dimensionality reduction, and report the resulting clusters as “cell types.” While this can provide a major step forward for areas of biology that are less studied, the immune system is among the most heavily studied and characterized aspects of human biology and it is important to interpret new technologies in the context of existing knowledge. Further, many biological processes are continuous and fluid, and thus it may not be appropriate or possible to assign cells to mutually exclusive labels.[11–14]

In this article, we introduce the Rhesus Immune Reference Atlas (RIRA), an scRNA-seq dataset composed of 426,664 cells from seven different tissues from 47 rhesus macaques (RMs), offering a comprehensive view of immune cell transcriptional diversity across seven distinct tissues in this important preclinical model species. This represents the first RM single-cell atlas and the first immune-focused atlas for any macaque species. We synthesize multiple sources of ground truth, including surface protein, sorted reference cells, and TCR data to anchor our cell type labels to established immunologic definitions. At the coarse cell-type level, we demonstrate transcriptional similarity (e.g., clustering) is sufficient to reliably delineate cell types. We validated RNA-centric marker definitions of these populations, which augment established surface protein definitions.

While transcriptional profiles and unsupervised clustering are robust at differentiating highly dissimilar cell types, we demonstrate that transcriptional similarity is not a reliable measure of functional similarity at finer resolutions, especially within T cells. Many functionally relevant T cell subsets lack differentiating RNA markers, and we demonstrate that the biological processes that dominate unsupervised clustering do not always align with established functional and lineage definitions. This outcome should not be surprising based on our knowledge of T cell biology, which involves highly plastic cell states and sharing of gene programs between distinct lineages; however, it is important to carefully understand the implications of this when interpreting unsupervised analyses.[11–14] Finally, we empirically identified sets of diagnostic gene modules with robust expression and high discriminatory power between subsets. We show that directly scoring cells for modules of known function, rather than relying on unsupervised analysis to discover them anew with each experiment, can complement unsupervised analysis by providing a consistent and stable definition of a biological process.

RESULTS

Selection of samples, quality control, and coarse cell type assignment

To provide a broad survey of immune cells, we generated single-cell suspensions from seven distinct tissues from 47 clinically healthy RMs, ages 2–7 (Figure 1A and Table S1). Data were multiplexed using lipid-based cell hashing and labeled with CITE-seq antibodies to measure surface protein (Table S2). Cells were processed using the 10X Genomics 5′ capture method, followed by a multi-step quality control and filtering process, described in detail in the STAR Methods section.

A total of 596,848 cells passed the primary quality control steps. We then performed dimensionality reduction (PCA/UMAP) using Seurat (Figure 1B).[6] We performed a two-phase approach to assign cells to coarse cell types. First, we scored cells for enrichment of gene modules composed of marker genes for major cell types, using rhesus macaque-adapted modules and the scGate R package (Figure 1C and Table S3). The rationale of this step is that high-level immune subsets each have well-defined marker genes and represent mutually exclusive lineages. While this is sufficient to categorize many cells, 17.9% of cells were not confidently assigned. This is not surprising, since this approach only considers a handful of markers in the classification, rather than utilizing the entire transcriptome. To build an improved classifier, we subset the data to cells with high-confidence assignment using scGate, down-sampled to achieve approximately equal numbers of cells per population, and used these data to build a ground-truth dataset.[15] We used these data to train a machine-learning classifier, which can utilize the full transcriptome in the resulting model, using CellTypist

CellPress
OPEN ACCESS

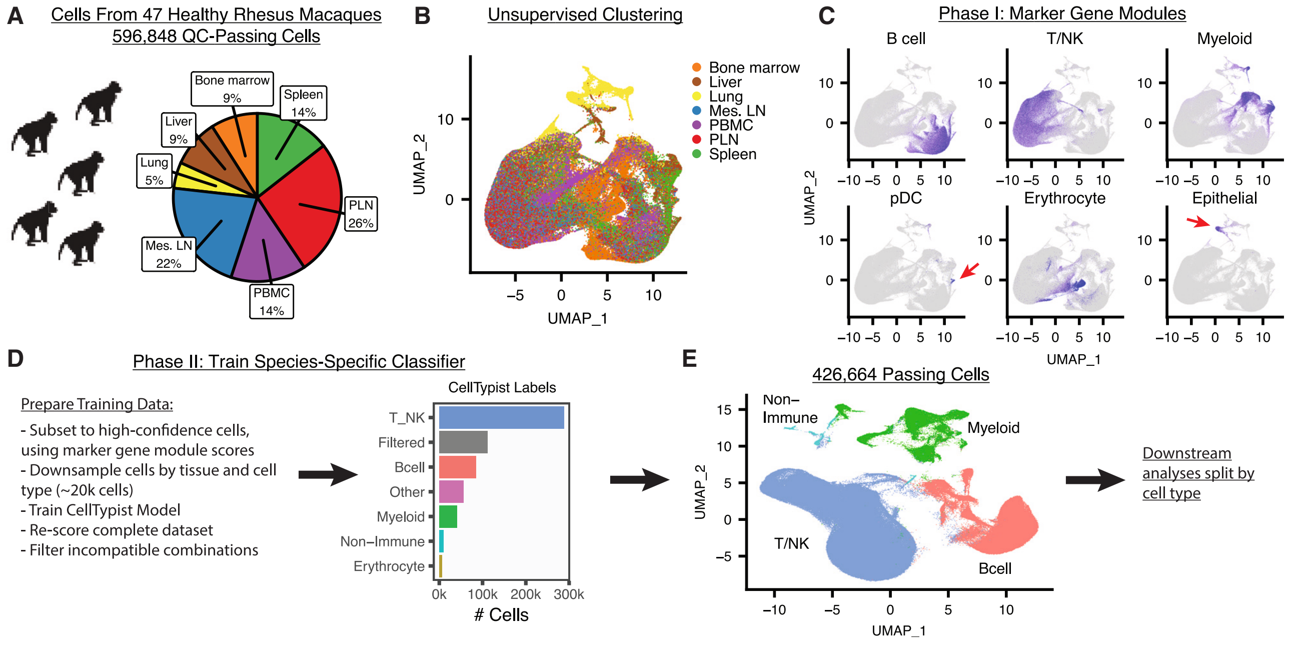

Figure 1. Overview of RIRA data and strategy for coarse cell type calling

(A) The pie chart summarizes the input cells and tissues.

(B) The UMAP plot displays unsupervised clustering of the preliminary data, colored by tissue.

(C) As a first step toward identifying cell types, cells were scored for gene modules consisting of canonical lineage marker genes (listed in Table S3). The panel of UMAP plots is colored according to enrichment for the indicated gene modules.

(D) To increase accuracy of cell type scoring, we subset cells from (C) with high-confidence calls, and down-sampled to create a training dataset. This training dataset was used to train a CellTypist classification model. The resulting model was used to label the entire dataset, with the results illustrated in the bar plot.

(E) After removal of ambiguous and filtered cells, PCA/UMAP was repeated, and the resulting projection is shown, colored by coarse cell type.

(Figure 1D).[16] We then scored the entire RIRA dataset, providing confident labels for 429,668 cells. As a secondary filter, we removed cells with incompatible cell types, such as those scoring highly as both a B and a T cell or T cell and erythrocyte, which likely represent doublets (see Table S4). A total of 426,664 cells remained (Figure 1E). We then proceeded to split the data by coarse cell type (e.g., myeloid cells, B cells, and T/natural killer [NK] cells) and to analyze each subset separately. The macaque cell type classification model and re-usable functions to perform and visualize the scoring/filtering used are incorporated into the RIRA R package (see STAR Methods).

For each coarse cell type, we performed unsupervised analysis of the transcriptional landscape, with the goals of (1) characterizing the major transcriptional subsets within each cell type, (2) defining labels for these populations that are grounded in established immune definitions using canonical marker genes and surface protein data, (3) identifying RNA features that correlate with canonical surface markers but provide higher diagnostic value in scRNA-seq data, and (4) evaluating the concordance between the dominant sources of transcriptional variation and established immunologic cell definitions and identifying biological programs that are orthogonal and confounding toward established functional distinctions.

Myeloid cell transcriptional diversity

The dataset contained 41,199 myeloid cells. After re-processing, unsupervised clustering identified 19 transcriptional clusters (Figure 2A). We compared the cluster-defining markers against canonical lineage markers, using both protein and RNA data, grouping cells into 14 types/states (Figure 2B). Within myeloid cells, there is generally concordance between the pattern of clustering and canonical immune cell types; however, the RNA features that dominate the pattern of clustering frequently differ from canonical protein markers, and many canonical surface protein markers perform poorly when evaluated by scRNA-seq. The panel of discriminatory RNA features we identified is important because canonical protein-based lineage markers are not always reliable when measured by RNA expression, and we identify highly informative RNA markers that are not well characterized as protein markers (Figure 2C).

Monocyte lineages

Within the myeloid cells, we identified clusters corresponding to monocytes, with separation of CD14^{+} classical monocytes from CD16^{+} non-classical monocytes (Figures 2B and 2C). While classical monocytes expressed CD14 protein and RNA, expression of versican (*VCAN*), an extracellular matrix proteoglycan, provided a more robust and specific marker.[17] Likewise, non-classical monocytes expressed both protein and RNA for the canonical marker CD16 (*FCGR3*); however, additional RNA markers such as *CX3CR1* and CD11c (*ITGAX*) were more specific in scRNA-seq data. Both lineages were enriched in peripheral blood mononuclear cells (PBMCs), as expected, although CD16^{+} non-classical monocytes were also present in large numbers in lung. Finally, we detected a subset of monocytes,

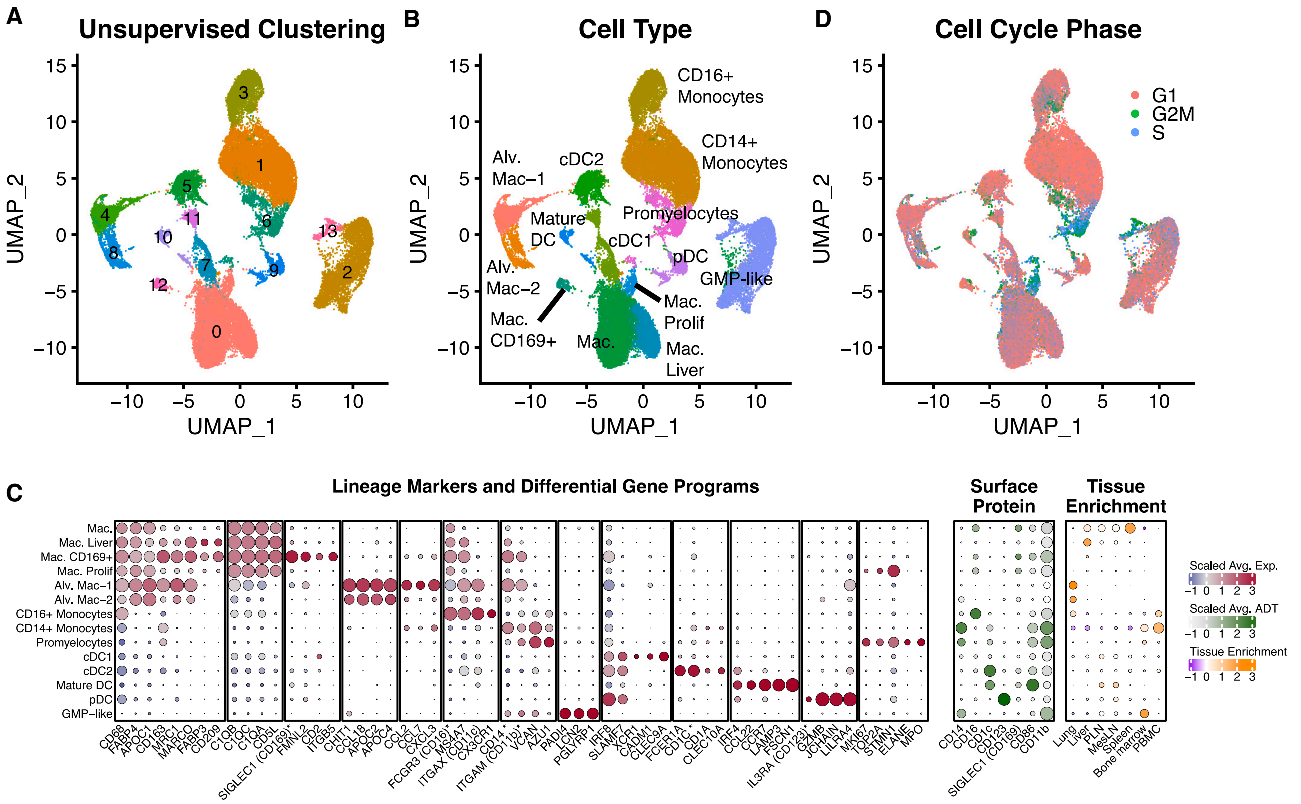

Figure 2. Phenotypic characterization of RIRA myeloid cells

(A) UMAP representation of the RIRA myeloid cell subset. Cells are colored by unsupervised clustering (Louvain).

(B) Identical UMAP to (A), colored by phenotypic labels determined using differential gene expression and canonical marker genes.

(C) The leftmost dot plot displays the top differentially expressed genes between the phenotypic classes shown in (B). The center dot plot displays surface protein expression for each group, and the rightmost dot plot displays tissue enrichment. Color scales correspond to column-scaled mean library-size-normalized RNA counts (RNA markers), column-scaled mean centered log ratio (CLR) normalized antibody-derived tag (ADT) counts (labeled surface protein), and linearly scaled chi-squared standardized residuals (tissue enrichment). Dot sizes correspond to fraction of cells expressing marker genes (RNA markers), fraction of cells positive for ADTs (surface protein), and fractional composition of tissues (tissue enrichment). Asterisks next to RNA feature names indicate the gene is also present in the surface protein section.

(D) Identical UMAP plot to (A), colored based on cell-cycle phase.

which are proliferative (defined using cell cycle algorithms and *MKI67* expression) and enriched in bone marrow, consistent with precursor promyelocytes, expressing *ELANE* and *MPO* (Figure 2D).[18]

Macrophage lineages

There were multiple macrophages clusters, with shared profiles, including expression of *CD68* (a common lineage marker), fatty-acid-binding proteins (*FAPB4*), and apolipoprotein expression (*APOC1*). We also identified multiple distinct macrophage subsets that highlight tissue specialization. Consistent with literature about the importance of lipid metabolism and fatty-acid-binding proteins in macrophage inflammatory and metabolic pathways, our data show that fatty-acid-binding protein and apolipoprotein expression provide strong discriminatory signals across macrophage subsets.[19] Lung alveolar macrophages (AMs), which are important in the response to pathogens, were among the most distinct. AMs are characterized by lack of complement expression (with complement genes being strong positive markers for other macrophages) and expression of the immunomodulatory gene *CHIT1* and apolipoprotein genes (*APOC2/APOC4*). Two major subsets of AMs were detected, with one characterized by gain of expression for *CCL2*, *CCL7*, and *CXCL3*. This unique inflammatory cytokine profile has been described as specific to lung-resident AMs, relative to monocyte-derived macrophages.[20] Another macrophage subset was highly enriched in the liver, characterized primarily by expression of DC-SIGN (*CD209*) and *FABP3*. The largest subset was enriched in secondary lymphoid tissues (spleen and lymph nodes), although this had few strong positive markers and was mostly defined by lack of the tissue-specific profiles listed above. A small cluster was identified, characterized by high RNA expression of *SIGLEC1* (CD169) and *FMNL2*, a gene involved in cytokinesis and cell polarity. CD169 provides an example of strong discordance between RNA and protein expression, with multiple macrophage subsets expressing CD169 protein, while *CD169* RNA provides a very specific marker for this small subset. While rare, the CD169$^+$ macrophages are noteworthy because CD169$^+$ macrophages have important roles in the defense against viral

and bacterial pathogens.[21] CD169$^+$ macrophages capture intact antigen and either present it directly or transfer immune complexes to dendritic cells (DCs).[22,23] Consistent with a role in cross-presentation and DC interactions, this subset upregulated DC-SIGN (*CD209*). Finally, a small cluster of proliferating macrophages was detected.

Plasmacytoid DCs

DCs are hematopoietic cells primarily known as antigen-presenting cells. They have several well-defined lineages, including classic and plasmacytoid lineages and sub-lineages within the classic DC subset. Plasmacytoid DCs (pDCs) have specialized functional properties, including secretion of type I interferons.[24] Consistent with this specialization, we identified a transcriptional cluster confirmed as pDCs based on protein staining for canonical DC surface marker CD123 (Figure 2C).[25] As with many surface markers, RNA detection of CD123 (*IL3RA*) was much less robust than protein; however, we identified several additional genes with pDCs-specific expression, including granzyme B (*GZMB*), *JCHAIN*, and *LILRA4*.

Conventional/classic DCs

Conventional or classical DCs (cDCs) can be divided into sub-lineages. The cDC1 lineage is thought to primarily function in cross-presentation of antigens to CD8$^+$ T cells, characterized by *XCR1*, *CADM1*, and *CLEC9A*.[26] The cDC2 lineage is thought to present antigen to CD4$^+$ T cells and is characterized by *CD1c*, Fc epsilon receptor (*FCER1A*), and *CLEC10A*.[26] We identified two clusters that strongly matched these profiles (Figure 2C). We also identified a third cluster that lacked the canonical markers of cDC1 and cDC2; however, it expressed *CCR7*, *LAMP3*, *CCL22*, and *FSCN1*. Expression of *CCR7* and *LAMP3* is consistent with activated, mature DCs with lymph node migratory potential.[27] The mature DC population was enriched in lymph nodes (Figure 2C). This subset has also been termed DC3 or mregDC in publications, defined as cDCs lacking conventional cDC1/cDC2 markers but expressing this activation-associated program.[28,29]

Granulocyte precursor cells

The methods used to isolate mononuclear cells and droplet-based scRNA-seq will select against granulocytes, and we did not detect significant numbers of mature neutrophils or other granulocyte cells in our dataset.[30] Nonetheless, we identified a population consistent with granulocyte monocyte progenitor (GMP) cells that was highly enriched in the bone marrow. These cells were defined by expression of the neutrophil lineage markers (*PAIDI4*), lipocalin-2 (*LCN2*), and peptidoglycan recognition protein 1 (*PGLYRP1*), consistent with prior publications.[18,31,32]

Collectively, these data demonstrate that, when dominant scRNA-seq analysis methods are applied to RM myeloid cells, at least in clinically healthy animals, the resulting clusters generally align with established immune cell types. While RNA encoding many canonical surface protein markers is reliably detected in scRNA-seq data, alternate genes are often more specific or robust as lineage markers when measured by RNA. These panels of markers and curated reference profiles provide an important tool for the interpretation of macaque scRNA-seq data.

B cell transcriptional diversity

Our dataset contained 89,788 B cells, which formed eight unsupervised clusters (Figure 3A). These were grouped into five subsets based on surface protein and expression of canonical markers (Figure 3B). Cell proliferation was a major driver of transcriptomic diversity, especially within pre-B and germinal center (GC) subsets (Figure 3C). Pre-B cells, GC cells, and plasma cells each formed a clearly distinct group, and differential gene expression revealed sets of highly discriminatory RNA markers for each (Figure 3D). Plasma cells, which have highly specialized function and morphology, were characterized by expression of *JCHAIN*, immunoglobulin heavy chain (*IGHA1*), and transcription factor X-box binding protein 1 (*XBP1*), which is important for plasma cell survival.[33] GC cells were characterized most robustly by expression of *RGS13*, a regulator of CXC chemokine responsiveness, transcription factor *MEF2B*, and activation-induced deaminase (*AIDCA*), all of which are consistent with published features of GC cells.[34–36] Pre-B cells expressed V-set pre-B surrogate light chain 1 (*VPREB1*) and the transcription factor *SOX*, both of which have been reported as markers of immature human B cells.[37] Pre-B cells were enriched in the bone marrow and were highly proliferative (Figures 3C and 3D). The largest subset contained mature B cells, with strong expression of *MS4A1* (CD20) and CD20 protein staining (Figure 3D). As with the myeloid data, the significance of this observation is that these distinct cell types have highly divergent transcriptional profiles, with multiple highly discriminatory markers, and unsupervised clustering is therefore expected to reliably discriminate between them.

Within the mature B cell cluster, we identified transcriptionally distinct sub-populations; however, most of these markers have continuous expression patterns and do not provide clear-cut subset definitions. *CD40* was among the strongest differentially expressed genes. The *CD40*$^-$ B cells, which are enriched in the PBMCs, have transcriptomic profiles consistent with published profiles of innate-like B cells, including reduced *CD40* and *CD69* and higher *CD1c* relative to classical low-IgM-expressing mature B cells, as well as expression of *AHNAK*.[38–41] The genes with the most robust expression, as measured by scRNA-seq, were *AHNAK*, *CD1C*, and *SOX5* (Figure 3E). Within the *CD40*$^+$ mature B cell population, we identified a cell consistent with both memory and naive B cells.[42–48] *PLAC8* has been used as an scRNA-seq marker for memory B cells in mice, *CR2* is a marker of human memory B cells, *LY86* has been used as an scRNA-seq marker for mature B cells in mice, and *CD44* has been used as an scRNA-seq marker for identifying different memory B cell populations in mice.[42–45] Expression of these memory B cell markers is significantly enriched in cluster 3, as shown in Figure 3F. *TCL1A* has been used as an scRNA-seq marker for naive B cells in human, *FCER2* has been used as an RNA-seq marker for human naive B cells, and CXCR4 has been used as a flow cytometry marker for naive B cells in RMs.[46–48] Expression of these naive B cell markers is enriched in cluster 0, as shown in the violin plot in Figure 3G. Within mature B cells, the sub-populations also do not form discrete clusters, but rather a phenotypic gradient, defined by the genes shown in the Figures 3F and 3G, respectively. We interpret the lack of clear lineage-defining markers to be the combination of two factors: (1) these represent continuous biological processes, and (2) the transcriptional footprint associated with naive-memory differentiation is relatively small as compared to all gene expression

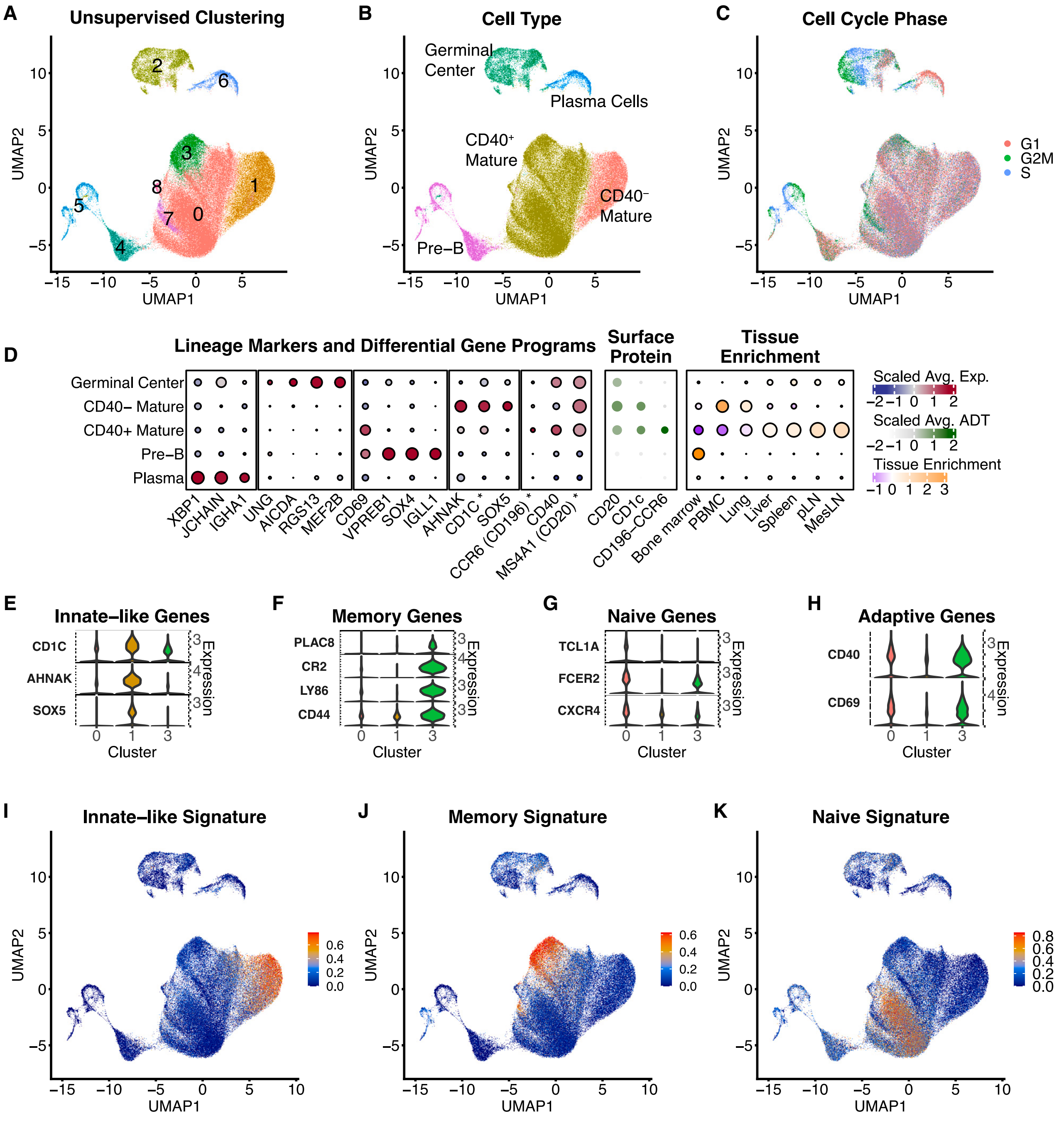

Figure 3. Phenotypic characterization of RIRA B cells

(A) UMAP representation of the RIRA B cell subset. Cells are colored by unsupervised clustering (Louvain).

(B) Identical UMAP to (A), colored by phenotypic labels determined using differential gene expression and canonical marker genes.

(C) Identical UMAP to (A), color based on cell-cycle phase.

(D) The leftmost dot plot displays the top differentially expressed genes between the phenotypic classes shown in (B). The center dot plot displays surface protein expression for each group, and the rightmost dot plot displays tissue enrichment. Color scales correspond to column-scaled mean library-size-normalized RNA counts (RNA markers), column-scaled mean CLR-normalized ADT counts (surface protein), and linearly scaled chi-squared standardized residuals (tissue enrichment). Dot sizes correspond to fraction of cells expressing marker genes (RNA markers), fraction of cells positive for ADTs (surface protein), and fractional composition of tissues (tissue enrichment).

(E–H) Violin plots show expression for a subset of genes whose expression differentiates sub-clusters within the primary cell types.

(I–K) All B cells were scored for enrichment of gene modules corresponding to the genes in (E)–(G). (I)–(K) contain identical UMAP plots to (A), where each is colored according to enrichment for the indicated gene module.

programs that impact the transcriptome of these cells. While there were few lineage-defining markers within mature B cells, we reasoned that scoring B cells for enrichment of multi-gene modules would provide a classification tool. Using the genes enriched in the innate-like, memory, and naive B cell populations (Figures 3E–3G), we generated per-cell enrichment scores with the UCell R package, which we demonstrate can enrich for these cell subsets, and provide transparent, transferable definitions of these cell subsets (Figures 3I–3K).[49]

Collectively, these data establish a reference for RM B cells and demonstrate that some B cell subtypes (pre-B, GC, plasma cells, and mature B cells) have highly distinct transcriptomic profiles that can be identified with a finite number of discriminatory RNA markers. As with many myeloid subsets, unsupervised clustering approaches should reliably discriminate between these B cell types. In contrast, while subsets exist within the mature B cell subset, the transcriptomes of these cells lacked clear lineage-defining markers. We nonetheless identified minimal, diagnostic gene modules that robustly enrich for these subsets. These reductionist scores can categorize cells without reliance on unsupervised clustering and can be easily documented and applied consistently across laboratories, providing many of the advantages of hierarchical marker-based gating. A continuous score, as opposed to assigning cells to mutually exclusive labels, as many classifiers do, may be a more appropriate way to represent continuous biological processes like memory differentiation.

T cell and NK transcriptional diversity

We next examined the 290,113 T cells and NK cells. While T and NK cells are distinct subsets with substantial differences in modes of antigenic stimulation, they share many effector functions and can have high transcriptional similarity.[6,50] Individual T cells undergo somatic rearrangement during thymic maturation to generate a unique a TCR, which mediates antigen recognition and is the basis of cellular adaptive memory. In contrast, NK cells and the related innate lymphoid cells (ILCs) recognize antigen through collective signaling of multiple families of germline-encoded receptors.[51] T cells are further sub-divided based on expression of co-receptors CD8 and CD4, which delineate cells recognizing antigen presented through the MHC-I and MHC-II systems, respectively. There are many functionally distinct subsets within T cells, including a well-established differentiation from naive into memory cells and separation of $\alpha\beta$ and $\gamma\delta$ T cells, two separate lineages that encode distinct TCR receptor chains and have different modes of antigen recognition.[52–54]

We began by contrasting the transcriptional clusters identified by unsupervised analysis (PCA/UMAP) with canonical lineages and differentiation states as well as modes of antigen recognition. We assigned labels to the transcriptional clusters based on gene expression (Figures 4A and 4B) and surface protein (Figures 4C–4E), with key markers shown in Figure 4D. An additional source of ground truth is TCR data (through V/J sequencing), which can categorize cells as $\alpha\beta$ TCR$^+$, $\gamma\delta$ TCR$^+$, and TCR$^-$ (Figure 4F). Importantly, the labels assigned to clusters are based on the dominant transcriptional characteristics of each cluster; however, we will demonstrate that most of these clusters are heterogeneous, containing a mixture of cells with important functional distinctions. This indicates that the gene expression programs that dominate unsupervised clustering of T and NK cells are often at odds with established functional classifications, and there is considerable sharing of gene programs between functionally distinct subsets. This complexity is likely exacerbated by the fact that many differentiation events in T cell maturation are continuous and there is considerable plasticity within T cell subsets, implying that cells can shift between different patterns of gene expression programs.[11–14] These observations are consistent with our understanding of T and NK cell biology; however, they have important implications for the interpretation of scRNA-seq data.

Limitations of standard lineage markers in T and NK cell scRNA data

We began by inspecting RNA expression for key lineage markers: CD3, CD4, CD8A, and CD8B (Figures 4C and 4D, left). With limited exceptions, RNA expression of these markers was not robust (Figure 4D, dot size). Detection of RNA for CD3 (genes *CD3D*, *CD3E*, and *CD3G*) was the most reliable, with clear differential expression between clusters dominated by T cells and the cluster dominated by NK cells. *CD8A* and *CD8B* RNA expression was relatively robust in naive T cells but less consistent in more differentiated T cells, suggesting that *CD8* RNA expression is downregulated as the cells undergo memory/effector differentiation. *CD4* RNA expression was extremely poor in all subsets, an observation consistent with prior publications and in stark contrast to CD4 surface protein (Figure 4C).[5] These data underscore the fact that cellular profiles measured by scRNA-seq will have different characteristics from protein, and classification of scRNA-seq profiles cannot rely on single marker genes. Perhaps more importantly, while unsupervised clustering largely separated CD3$^+$ T cells from CD3$^-$ NKs and largely separated CD8$^+$ and CD4$^+$ T cells, these clusters are rarely pure. For example, while we identified two clusters with highly cytotoxic cells, one enriched for NKs (defined as CD3 negative and TCR negative) and one enriched for T cells, both contained a mixture of cell types including $\alpha\beta$ T cells, $\gamma\delta$ T cells, and NKs (Figure 4F). While it is possible that, at a higher cluster resolution, there might be a separation of NKs and true T cells, this emphasizes the danger in relying on transcriptional similarity to distinguish between functionally distinct subsets. Within the cytotoxic T effector memory-like (T_{EM}-like) and central memory-like (T_{CM}-like) clusters, while almost no *CD4* RNA-positive cells were detected, more than 10% of the cells were positive for CD4 surface protein (Figure 4E). While the existence of CD4$^+$ cytotoxic T cells is known,[55] the transcriptomes of these cells would be nearly indistinguishable from CD8$^+$ cytotoxic T cells without surface protein staining. This outcome is not a failure of unsupervised clustering, but rather it highlights a fundamental difference between algorithms designed to separate cells by global transcriptional variation and gating using curated marker panels. This also suggests that cytotoxic differentiation exerts a strong footprint on T/NK cell transcriptomes.

Naive and memory T cell differentiation

T cells undergo a differentiation process beginning with naive T cells that are selected in the thymus, after which they circulate until they encounter antigen, whereby they can undergo selection to differentiate into effector and memory populations.[56] Human naive T cells are classically separated from memory T cells

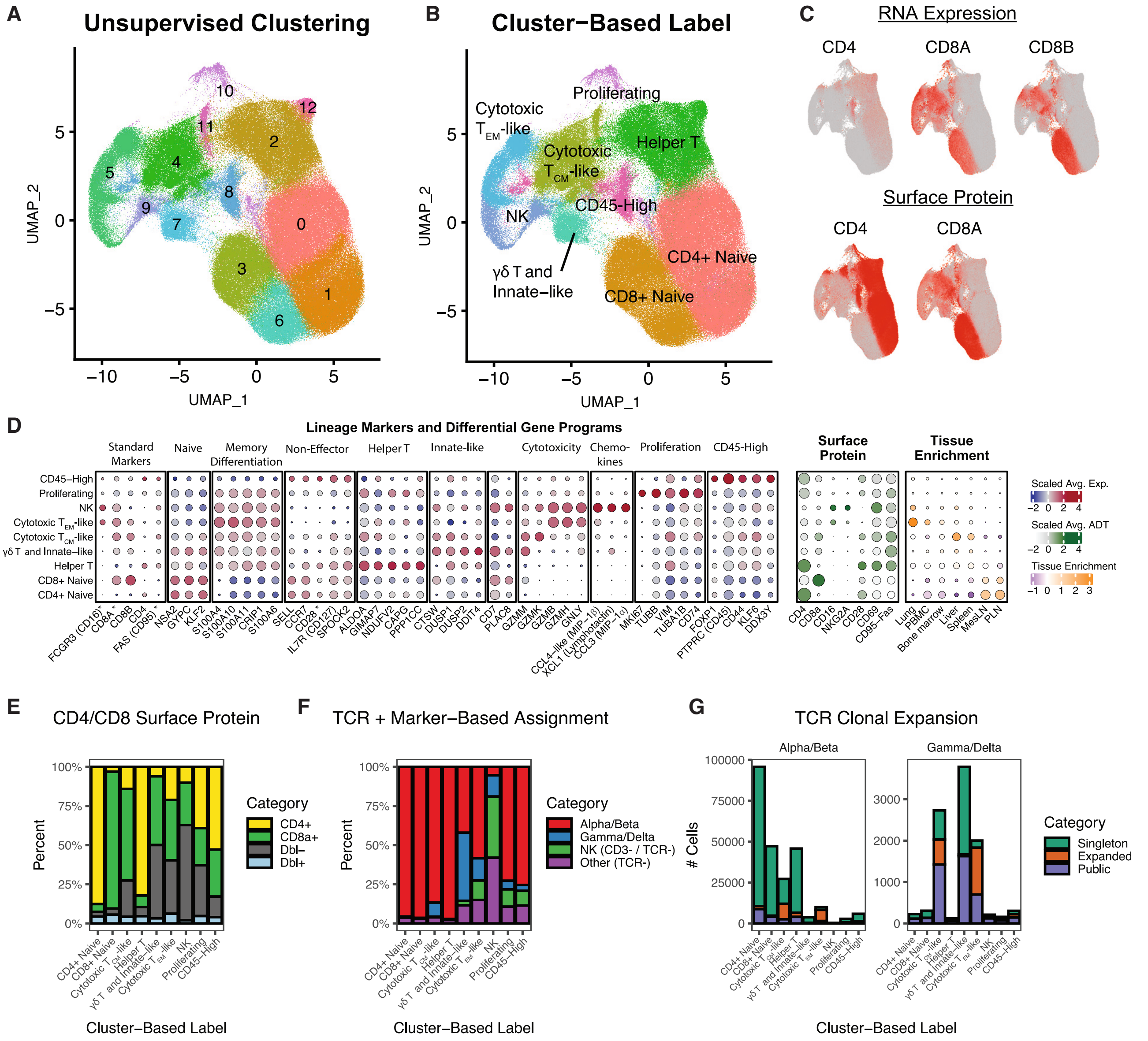

Figure 4. Phenotypic characterization of RIRA T and NK cells

(A) UMAP representation of the RIRA T and NK cell subset. Cells are colored by unsupervised clustering (Louvain).

(B) Identical UMAP to (A), colored by phenotypic labels determined using differential gene expression and canonical marker genes.

(C) Similar UMAP plots to (A), colored by the indicated RNA features (top row), or surface protein stain (bottom row). These panels illustrate the difference between RNA and protein detection, highlighting the limitation for CD4 RNA.

(D) The leftmost dot plot displays the top differentially expressed genes between the phenotypic classes shown in (B), along with key marker genes. The center dot plot displays surface protein expression for each group, and the rightmost dot plot displays tissue enrichment. Color scales correspond to column-scaled mean library-size-normalized RNA counts (RNA markers), column-scaled mean CLR-normalized ADT counts (surface protein), and linearly scaled chi-squared standardized residuals (tissue enrichment). Dot sizes correspond to fraction of cells expressing marker genes (RNA markers), fraction of cells positive for ADTs (surface protein), and fractional composition of tissues (tissue enrichment).

(E) The bar plot shows the percentage of cells from each phenotypic group defined in (B) that are positive for CD4 and/or CD8a protein, demonstrating that most cluster-based groups are heterogeneous, except for naive T cells.

(F) A similar bar plot to (E), colored according to a categorization based on a combination of (1) presence of αβ and/or γδ TCR, and (2) presence of CD3 and/or CD16 RNA. As with (E), this illustrates that most cluster-based groups contain a mixture of functional subtypes.

(G) The bar plots summarize TCR clonotype data per cluster-based group based on whether the clonotype is a “singleton” (detected in a single cell), “expanded” (detected in multiple cells in a single RM), or “public” (detected in more than one RM). Data are separated into alpha/beta and gamma/delta T cells.

based on CD45RA/RO protein; however, there are species-specific differences, and CD95, CD28, and CCR7 are commonly used to define memory subsets in RMs.[57,58] In our analysis, naive T cells largely segregated from memory cells in unsupervised clustering (Figure 4B). The clusters labeled as naive are supported by a lack of CD95/FAS (RNA and protein), expression of lymph node homing markers *CCR7* and *SELL* (CD62L; L-selectin), and strong enrichment in lymph nodes.[58] These clusters were almost exclusively $\alpha\beta$ T cells, and the vast majority of the TCR sequences detected in these cells were singletons, which is expected for cells that have not undergone antigenic expansion (Figures 4F and 4G). There were very few RNA markers that clearly differentiated naive and memory T cells. CD95/FAS RNA was negative in most cells, which could reflect RNA/protein differences (Figure 2D). A handful of genes were enriched in naive T cells, including *KLF2*, *NSA2*, and *GYPC*; however, these are not exclusively expressed in naive T cells (Figure 4D). Interestingly, we identified multiple members of the S100A protein family, which are involved in calcium binding, that provided strong positive markers for memory T cell differentiation (Figure 4D). While these are not common surface markers, they are involved in T cell memory.[59] These data demonstrate that naive-to-memory differentiation is a significant driver of T cell transcriptional variance; however, it is a continuous rather than discrete process and the transcriptional features associated with memory differentiation differ from classic protein markers.

Cytotoxic vs. helper T cells and CD4/CD8 co-receptor expression

T cells are classically separated into cytotoxic and helper cells, which act through either cytotoxicity or the secretion of factors to modulate other immune cells.[60] T cells also are commonly sub-divided by surface protein expression of the co-receptors CD8 and CD4. Cytotoxic T cells are more likely to express CD8, with non-cytotoxic helper T cells more likely to express CD4, and thus cytotoxicity and co-receptor usage can be conflated.[61] Genes involved in cytotoxicity were major drivers of transcriptional variance, and we identified clusters enriched for T_{CM}-like and T_{EM}-like cells expressing cytotoxic markers, including multiple granzyme genes and *NKG7* (Figure 4D). NK cells—not surprising based on function—clustered with highly cytotoxic T cells. Within cytotoxic T and NK cells, the pattern of granzyme gene expression provided an informative marker of differentiation. We detected granzyme M expression in naive $CD8^+$ T cells, granzymes M and K in cytotoxic T_{CM}-like cells, and some NK cells, with granzymes B and H expressed in cytotoxic T_{CM}-like cells and the remaining NK cells (Figure 4D). As noted above, a significant number of T cells with cytotoxic profiles were positive for CD4 surface protein, even though *CD4* RNA is undetectable from most of these cells. This is an important caveat because unsupervised clustering of scRNA-seq data will tend to separate cytotoxic and helper T cells, while flow cytometry typically separates T cells based on CD4/CD8 co-receptor usage. These two definitions have a high degree of overlap but will systematically differ.

There were far fewer positive markers of helper T cells, suggesting that, at least prior to stimulation, acquisition of memory and lack of cytotoxic differentiation is a defining feature of the helper T cell transcriptome. The primary positive marker was fructose-bisphosphate aldolase A (*ALDOA*), an enzyme involved in glycolysis.[62] While this cluster is likely composed of multiple subtypes, including T helper (Th)1, Th2, Th17, Tfh, and regulatory T (Treg) populations, the canonical markers used to differentiate these cells tended to be poorly detected at the RNA level, at least in resting cells, as has been previously reported.[63] Their distinct functional properties may only become apparent after stimulation or using measurements of transcriptional potential like assay for transposase-accessible chromatin with sequencing (ATAC-seq).

Phenotypic and functional heterogeneity within most transcriptional clusters

Except for the naive T cell clusters, all clusters intermingled functionally distinct subsets. In addition to the examples discussed above for T and NK cells and cytotoxic $CD4^+$ T cells, we identified a cluster enriched for both $\gamma\delta$ T cells and innate-like $\alpha\beta$ T cells. These cells expressed memory differentiation markers with limited markers of cytotoxicity, suggesting these cells share many transcriptional programs, even though these cells represent distinct lineages with distinct antigenic recognition. We further identified a highly distinct transcriptional cluster of proliferating cells. These clusters contained cells with a mixture of T and NK cell lineage markers, mixture of CD4 and CD8 surface protein, and mixture of naive/memory differentiation states. We additionally identified a cluster of cells characterized by high CD45 (*PTPRC*) expression, with low RNA levels. These cells were also enriched for *FOXP1*, a gene primarily associated with quiescence, activation marker *CD44*, and transcription factor *KLF6*.[64] The observation highlights the fact that gene programs that provide the great transcriptional variance in scRNA-seq data are not necessarily aligned with established functional distinctions of immune cells and represent an important consideration for any publications that attempt to use clustering as a guide for T and NK cell classification.

Sorted reference T cell data provide additional ground truth

Our data demonstrate that the biological process of naive-to-memory differentiation is a major driver of transcriptional variation in T cells. To more precisely characterize these populations, we collected an independent reference dataset using sorted peripheral T cells from six independent clinically healthy donors. We sorted the following subsets: $CD4^+$ naive T cells ($CD3^+$/$CD4^+$/$CD95^-$/$CD28^+$), $CD8^+$ naive T cells (T_N, $CD3^+$/$CD8^+$/$CD95^-$/$CD28^+$), $CD4^+$ T central memory/$CD4^+$ transitional memory (T_{CM}/T_{TM}, $CD3^+$/$CD4^+$/$CD95^+$/$CD28^+$), $CD8^+$ T_{CM}/T_{TM} ($CD3^+$/$CD8^+$/$CD95^+$/$CD28^+$), $CD4^+$ T effector memory (T_{EM}, $CD3^+$/$CD4^+$/$CD95^+$/$CD28^-$), $CD8^+$ T_{EM} ($CD3^+$/$CD8^+$/$CD95^+$/$CD28^-$), and $\gamma\delta$ T cells ($CD3^+$/$CD4^-$/$\gamma\delta^+$).

When we performed dimensionality reduction using standard pipelines, the resulting clusters tended to separate naive from central memory and effector memory cells, although almost no clusters corresponded to a single population (Figure 5A). This is consistent with our full dataset (Figure 4). Nonetheless, to rule out the possibility that alternative data processing would generate clusters that more robustly map to reference populations, we processed these data using both the Seurat and ScanPy pipelines, followed by unsupervised clustering using

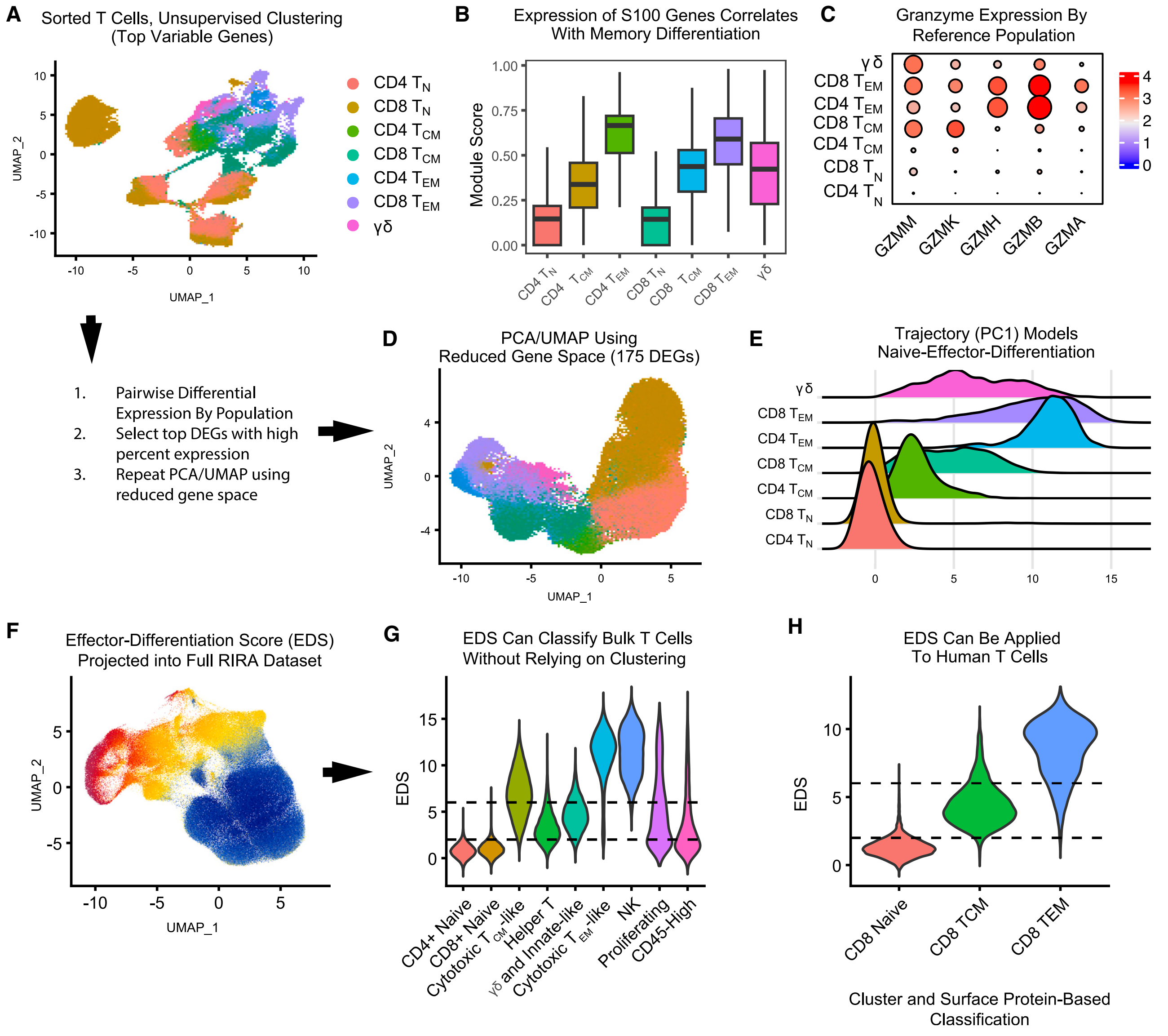

Figure 5. Sorted reference T cell data and simplified statistic to model naive-to-memory differentiation

Reference populations of T cells were fluorescence-activated cell sorting (FACS) sorted from PBMCs, followed by scRNA-seq.

(A) The UMAP plot displays dimensionality reduction of 56,637 sorted reference T cells, colored by population. Dimensionality reduction was performed on the top 3,000 variable genes.

(B) Per-cell enrichment was calculated for a gene module consisting of S100A proteins. The boxplot displays the range of this score by reference population.

(C) The dot plot displays the pattern of granzyme expression within each reference population.

(D) Pairwise differential gene expression contrasts were performed by population to identify the top marker genes per population. The UMAP plot displays dimensionality reduction in this reduced gene space using the 175 top marker genes for these populations.

(E) PC1 from (D) was imputed into the sorted cells. The plot displays the range of these scores, illustrating the separation of the reference populations.

(F) To test the generalizability of this statistic, the same principal component was imputed into the full RIRA dataset. The UMAP plot displays this effector-differentiation score (EDS).

(G) The same scores as in (F) are displayed as a violin plot. The dotted lines illustrate representative thresholds that could be used to subset data to remove naive T cells or limit to effector-differentiated cells.

(H) The EDS was used to score peripheral T cells from a public human dataset, demonstrating applicability across species. Collectively, these data demonstrate that S100A proteins and granzyme expression have predictive power to separate T cell subsets and further show that the EDS, which was modeled using reference peripheral T cells, provides a transferable statistic to quantify naive-to-effector differentiation in T cell datasets.

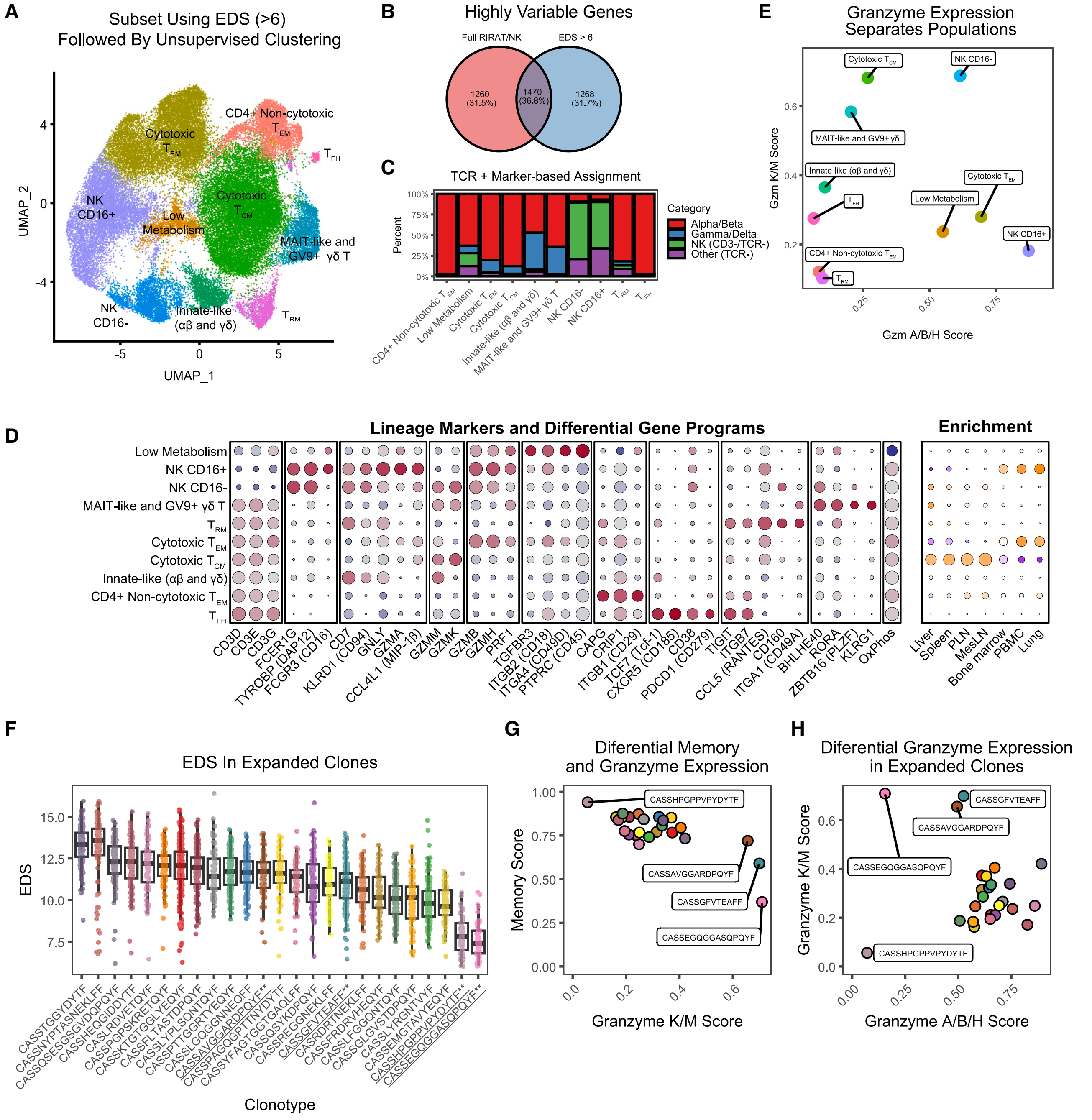

Figure 6. Analysis of effector-differentiated T and NK cells

(A) The UMAP plot displays dimensionality reduction of RIRA T/NK cells, after limiting to cells with EDS > 6.

(B) The Venn diagram shows the overlap between the top 3,000 variable genes, which is the input to PCA/UMAP, computed on the total RIRA T/NK dataset as compared to this subset. This highly different gene space is one of the reasons for different pattern of clustering at this resolution.

(C) The bar plot summarizes each cluster/phenotype based on a combination of (1) presence of αβ and/or γδ TCR, and (2) presence of CD3 and/or CD16 RNA. This illustrates that many cluster-based groups contain a mixture of functional subtypes.

(D) The leftmost dot plot displays the top differentially expressed genes between the phenotypic classes shown in (A), along with key marker genes. The rightmost dot plot displays tissue enrichment. Color scales correspond to column-scaled mean library-size-normalized RNA counts (RNA markers) and linearly scaled chi-squared standardized residuals (tissue enrichment). Dot sizes correspond to fraction of cells expressing marker genes (RNA markers) and fractional composition of tissues (tissue enrichment).

(E) Cells were scored for enrichment of granzymes K/M and granzymes A/B/H using the UCell module. The graph displays the mean of each score per cluster, demonstrating that these modules have predictive power among many effector subsets.

(legend continued on next page)

multiple approaches (see supplemental information).[6,8] While different bioinformatic pipelines produced slightly different clustering patterns, all clusters intermingled cells from different populations. These analyses continue to demonstrate that the transcriptomic features that govern neighborhood clustering do not inherently align with canonical definitions of immune cell subsets.

These sorted data confirmed several observations from the full RIRA T and NK dataset. S100 protein expression correlated with memory differentiation in both $CD4^+$ and $CD8^+$ T cells (Figure 5B). We detected informative patterns of granzyme gene expression during the maturation of cytotoxic T cells, with $CD8^+$ T_N cells expressing *GZMM* alone, $CD8^+$ T_{CM}/T_{TM} expressing *GZMM* and *GMZK*, and $CD8^+$ T_{EM} primarily expressing *GZMB* and *GMZH*. $CD4^+$ T_{EM} showed similar granzyme expression to $CD8^+$ T_{EM}, while $CD4^+$ T_{CM}/T_{TM} showed little granzyme expression at all (Figure 5C). This suggests these gene modules are informative diagnostic tools to classify T and NK cells.

Independently modeling T cell memory differentiation

Standard scRNA-seq pipelines identify the top variable genes in a dataset and perform dimensionality reduction on these genes.[6,8] This reduced gene space is designed to capture signals with the largest variance in the data; however, this represents the integral of many concurrent biological processes, and this set of genes will vary based on the composition of the input data. We reasoned that a more focused approach could better model naive-memory differentiation. To identify the core genes that differentiate these subsets, we performed pairwise differential gene expression contrasts using the sorted data. To find the most robust marker genes, we filtered the differentially expressed gene set to include only those expressed in a high fraction of cells. We repeated PCA/UMAP using a reduced gene space limited to these markers (n = 175 genes; Table S5) on the reasoning that this would provide the optimal feature set to differentiate these populations. The resulting UMAP revealed a general axis separating T_N, T_{CM}/T_{TM}, and T_{EM} cells (Figure 5D). Most importantly, the gene trajectory produced by the first principal component (PC1) was effective at modeling naive-memory differentiation (Figure 5E; gene weights listed in Table S5). This is significant because this component provides a reductionist, standalone classification tool that could be applied to other datasets, providing a transparent and simple measurement of this important biological process. To demonstrate this, we took this component, which we termed the effector-differentiation score (EDS) and imputed this score into the full RIRA dataset (Figure 5F). We demonstrate that, despite being defined using peripheral T cells, the EDS effectively categorized cells from diverse tissues. We also applied the EDS to a human dataset containing reference T cell populations (defined by surface antibody staining) and determined that the EDS can also accurately categorize human cells (Figure 5H).[65] Unlike methods that require dimensionality reduction, the EDS provides a direct and reproducible measurement of this important biological property, facilitating uniform and consistent definitions of cell populations. While we believe that modeling naive-memory differentiation as a continuous variable most accurately represents the underlying biology, it is possible to use EDS-based cutoffs to categorize cells into discrete classes (Figures 5G and 5H, dotted lines). The ability to score scRNA-seq data using the EDS has been implemented in the RIRA R package (see STAR Methods).

The transcriptional landscape of effector-differentiated T and NK cells

We demonstrated that the EDS can be used to categorize the differentiation state of T cells in a manner that is deterministic and reproducible, in contrast to reliance on data-dependent analysis such as unsupervised clustering. One practical application for the EDS is to subset data in a consistent manner, akin to gating in flow cytometry. The effector-differentiated T and NK cells, in contrast to naive T cells, are licensed to respond to antigenic stimulation, and our analyses suggest there is greater heterogeneity within these cells. An unsupervised analysis limited to more effector-differentiated cells will therefore highlight differential gene programs specific to these cells and highlight different subsets within them.

We subset the entire T/NK dataset to cells with EDS > 6, a threshold selected to capture effector-differentiated cells (Figure 5G, top dotted line). Not surprisingly, PCA/UMAP performed on this focused dataset produced distinct patterns of clustering relative to bulk T and NK cells, highlighting distinct subsets (Figure 6A). A basic reason for this outcome is that the top variable genes within this more homogeneous effector-like subset differ from the top variable genes calculated on bulk T and NK cells (Figure 6B). We then contrasted each transcriptional cluster against known T and NK cell subsets. While most clusters can be connected to established T and NK cell subsets, these data demonstrate that effector functions, rather than conventional functional classifications, dominate the pattern of clustering (Figures 6C and 6D). This has important implications for the interpretation of scRNA-seq data. A description of each major subset is listed below.

NK cell subtypes

After sub-setting, unsupervised clustering provided a clearer separation between T and NK cells, with two clusters highly enriched for NK cells. Nonetheless, approximately 10% of each NK-enriched cluster consisted of highly cytotoxic T cells, further demonstrating these two cell types have highly similar transcriptomes and highlighting the pitfalls encountered when equating cluster and cell type (Figure 6C). Among the two NK subsets, one was primarily $CD16^+$ and one primarily $CD16^-$ (Figures 6A and 6D). The former is enriched in PBMC, while the latter is enriched in liver and spleen. These subsets are consistent with previous flow cytometry-based studies.[66] The $CD16^+$ NK cells are characterized by a more cytotoxic phenotype, lacking granzymes M and K, with increased granzyme B and H, and acquisition of granzyme A. Differential granzyme expression is

(F) The top 25 most expanded TCRβ clonotypes were selected. The boxplot displays the EDS of each cell. These data illustrate that most clones are highly effector differentiated, although two outliers exist. Clones labeled in (G) and (H) are underlined, and colors are preserved in (F)–(H).
(G) The plot displays the mean memory score (defined by S100A proteins) compared to the granzyme K/M score. Clones with atypical profiles are labeled.
(H) The plot displays the mean granzyme K/M and mean granzyme A/B/H score for each clone. As in (G), atypical clones are labeled.

significant because granzymes have robust RNA expression and provide a strong diagnostic signal. Scoring cells for enrichment of gene modules for either granzymes M/K or granzymes A/B/H is highly discriminatory at separating these subsets (Figure 6E).

Subsets within effector-differentiated T cells

In addition to NK-enriched clusters, we detected a cluster containing primarily $\alpha\beta$ T cells, characterized by granzyme B, granzyme H, and perforin, consistent with our sorted T_{EM} cells (Figure 5). A cluster of $\alpha\beta$ T cells enriched for granzymes M and K, consistent with T_{CM}/T_{TM}, was also identified, enriched in secondary lymphoid tissue and liver. Two clusters that intermingled $\gamma\delta$ and $\alpha\beta$ T cells were present. One cluster contained both MAIT-like $\alpha\beta$ T cells (TRAV1-2$^+$, *ZBTB16*/PLZF$^+$) and *GV9*$^+$ $\gamma\delta$ T cells. The co-clustering of MAIT-like $\alpha\beta$ T cells, which recognize microbial metabolites presented by MR1 and *GV9*$^+$ $\gamma\delta$ T cells, which are thought to recognize microbial phosphoantigens independent of MHC, is noteworthy.[67] These represent distinct lineages that diverged very early in cell maturation; however, the sharing of gene programs (shown by co-clustering) likely reflects similar effector functions. Their transcriptomes are characterized by a granzyme K/M-skewed profile (akin to T_{CM}/T_{TM} $\alpha\beta$ T cells); however, these cells actively expressed perforin, which distinguishes them from most cytotoxically differentiated T_{EM} cells and is consistent with a state licensed for rapid response.[68,69]

The majority of the remaining $\gamma\delta$ T cells co-clustered with a subset of $\alpha\beta$ T cells, which we termed innate-like T. These cells expressed *CD94* but very little granzyme or perforin; however, they had the highest granulysin expression among T cells. Because granulysin acts against membranes lacking cholesterol, such as bacteria, fungi, and other parasites, this is suggestive of cells tuned to fight microbial pathogens; however, granulysin can also act as an alarmin to recruit antigen-presenting cells.[70] This profile is consistent with features of ILCs, although the fact that the majority of cells in this cluster expressed TCRs suggests this is another instance where T/NK cells are clustered by convergent functional profiles rather than lineage or antigenic recognition.[71]

At this resolution, we also identified a cluster comprised primarily of effector CD4$^+$ T cells, characterized by low cytotoxicity and positive expression of *CRIP1*, *CAPG*, and *ITGB1*/CD29. These markers are noteworthy because *CD4* RNA expression is relatively poor, and these genes may provide a more robust signature to identify effector CD4$^+$ T cells. A discrete population of T follicular helper (T_{FH}) cells was detected, characterized by *CXCR5*, *TCF7* (Tcf-1, a marker of self-renewal), *TIGIT*, chemotactic receptor *GPR183*, *ITGB7* (integrin β7), *PDCD1* (PD-1), and *CD38*.[72] We also detected a cluster consistent with tissue resident memory (T_{RM}). While we did not detect expression of canonical protein T_{RM} markers such as CXCR6, they expressed *ITGA1* (CD49A), *CD160*, *TIGIT*, *ITGB7*, and notably had high levels of *CCL5* (RANTES) RNA.[73,74] The latter is notable since T_{RM} cells function as immediate responders to antigen, with RANTES being rapidly secreted.[75] Finally, a cluster was detected that shared features with the T_{EM}-like cluster but was defined by high cytotoxicity, low cellular metabolism, and reduced oxidative phosphorylation. These cells upregulated *ITGA4* (CD49D), *TGBFR3*, *CD38*, and the scaffold protein *AHNAK*. While our data cannot formally demonstrate this, this phenotype is consistent with antigen exposure and profiles reported for exhausted, terminal effector cells.[76,77]

Collectively, these data establish the dominant gene expression profiles that separate the T and NK cell landscape, as processed through standard scRNA-seq pipelines. These data demonstrate that effector differentiation programs, in particular cytotoxicity, are strong drivers of transcriptional diversity and these programs have a strong influence on the clustering of T and NK cells. As such, unsupervised clustering frequently produces clusters that contain cells with similar effector profiles but a mixture of distinct lineages and, perhaps most importantly, different modes of antigenic stimulation.

Direct measurement of functionally relevant gene programs complements unsupervised analysis

Our data demonstrate that, while unsupervised clustering can be a powerful tool to discover signals in high-dimensional data, it has important limitations. This clustering is, by design, driven by the genes with the largest impact on transcriptional variance of the input data. We demonstrated the fungibility and data dependency of clustering in our analyses (Figures 4, 5, and 6). We also empirically defined gene modules (granzyme patterns or S100 protein expression) and latent factors (the EDS) that correlate with T and NK cell differentiation and functional states. In fact, the pattern of granzyme K/M versus granzyme A/B/H expression by itself is informative at differentiating between many effector T and NK subsets (Figure 6E). This is significant because measurement of targeted gene modules, in contrast to unsupervised clustering, is impacted less by orthogonal gene programs and can be more easily reproduced across analyses and laboratories.

To further examine the utility of these targeted gene modules, we subset our data to include highly expanded T cell clones. Each clonotype is a biologically related unit of cells, with the TCR sequence conferring antigen specificity, and these clonotypes have putatively been expanded due to antigen exposure. We reasoned that directly scoring these clones for functionally relevant gene programs could provide information about the differentiation state of these cells. When we examined the EDS, which we validated as a measure of effector differentiation, all expanded T cell clonotypes were highly differentiated relative to bulk T cells; however, two clonotypes were markedly less differentiated (Figure 6F, right). When we examined granzyme and memory differentiation (S100 protein) modules, the majority of expanded clonotypes scored highly for memory differentiation and were highly skewed toward granzyme A/B/H, with low granzyme K/M, a profile consistent with T_{EM} cells. However, clonotypes CASSAVGGARDPQYF, CASSGFVTEAFF, and CASSEGQGGASQPQYF, which were each heavily enriched in the liver and spleen, were outliers. These clones have a granzyme K/M-skewed profile, consistent with cytotoxic T_{CM} cells. It is tempting to hypothesize these cells represent newly expanded memory; however, our data cannot confirm this. Nonetheless, our data demonstrate that clonal expansion of cells with this T_{CM}-like profile is relatively rare in clinically healthy RMs and provide a reference for future functional studies. Clone CASSHPGPPVPYDYTF, which has high memory differentiation but low granzyme scores (Figures 6G and 6H), was a CD4$^+$ clone found exclusively in the

lung. Because granzymes and cytotoxic differentiation have established functional properties, the lack of these features provides information about the function of this clone. As above, our data demonstrate that expansion of clones with this non-cytotoxic phenotype is rare in clinically healthy RMs. Collectively, these data provide an illustration of how curated gene sets, selected for robust detection by scRNA-seq platforms and for correlation to specific biological processes, can complement unsupervised analyses.

DISCUSSION

Here we present the first single-cell immune atlas for RMs, a major preclinical model organism. While RMs and humans are closely related, there are species-specific differences, and the generation of macaque-specific reference data have broad value for research conducted in any macaque species. We established a detailed reference of the transcriptomic landscape of major immune cell types, documenting the major sources of transcriptomic diversity across and within immune subsets. We used surface protein staining and canonical marker genes as ground truth to contrast the transcriptional landscape identified by scRNA-seq against established models of immune lineages and functional classes. While it is generally understood that surface marker definitions do not always align with scRNA-seq profiles, it is essential to thoroughly understand these differences to accurately interpret scRNA-seq data. At a coarse cell-type level, where cell types tend to be very distinct and non-overlapping, unsupervised clustering of scRNA-seq data will tend to produce clusters that unambiguously match conventional cell types (e.g., B cells and monocytes). The expression of marker genes is usually sufficient to differentiate these dissimilar cell types, although the specific genes that perform well in scRNA-seq data, empirically defined here, frequently differ from canonical markers. Machine-learning classifiers, which we generated here, also perform well.

At finer resolutions, the patterns of clustering based on scRNA-seq data are less likely to cleanly map to canonical functional subsets and can systematically intermingle functionally distinct subsets. This was particularly apparent within T and NK cells but was also true within mature B cells. We demonstrate that the biological processes of memory and cytotoxic differentiation are major drivers of T and NK cell transcriptional diversity; however, there is considerable sharing of gene programs between NK cells, $\alpha\beta$ T cells, and $\gamma\delta$ T cells. As a result, unsupervised clustering is more likely to segregate cells based on their effector profiles than canonical lineages. This could be argued as a feature of scRNA-seq analysis, since it allows for the identification of shared transcriptional programs; however, it highlights a danger in over-interpretation of the significance of clusters and reliance on the assumption that cells of a cluster share functional properties. These cell types each recognize antigen through distinct mechanisms, and thus conflating highly cytotoxic T cells with NK cells (even if they possess highly similar transcriptomes) is problematic. Another systematic difference in scRNA-seq data is the conflation of CD4/CD8 co-receptor expression and cytotoxic/helper T cells. The dominant gating strategy used in flow cytometry separates T cells based on $CD4^+$ and $CD8^+$ co-receptor protein expression. In contrast, we show that unsupervised analyses tend to segregate T cells based on cytotoxicity (or lack thereof), a state that is correlated with but not identical to CD4/CD8 expression. These observations are consistent with the underlying biology of these cells and the way dominant scRNA-seq analysis pipelines function; however, it is important for scientists to be aware of these systematic differences and incorporate them into the interpretation of scRNA-seq profiles. The gene programs that provide the most reliable signal for a given biological property are not necessarily identical to canonical protein markers. For example, we demonstrate that expression of S100A proteins, which are involved in calcium binding, are a much more reliable signature of T cell memory than genes encoding canonical protein markers.

Our data highlight a related challenge for scRNA-seq data: disentanglement of cell type and cell state. While highly dissimilar cell types tend to have strong differences, this is not always true for more similar cell types. Further, many differentiation states are known to be plastic, and the transcriptome of a cell is the integral of many concurrent biological processes. These have several important implications. First, it may not be desirable to assign cells, at least as some resolutions, to mutually exclusive cell types. Instead, it may be more biologically appropriate to identify and measure specific gene programs, such as lymph node or tissue homing, and to view these processes as continuous variables rather than discrete states. Further, while unsupervised, exploratory analyses are an excellent tool to discover these programs, the field needs to evolve beyond the need to "re-discover" these key programs anew with each experiment. The design of dominant scRNA-seq pipelines also means that even if two analyses detected the same biological programs, the manner in which they are measured is indirect and dataset dependent. Development of targeted, transparent, and reproducible approaches to measure key processes would facilitate consistency and interoperability across studies.

We used our data to identify diagnostic gene programs that measure T and NK cell differentiation states. Using sorted reference cells, we defined an EDS, which uses a discrete gene set to model naive-to-effector memory differentiation in human and macaque T cells, a well-defined biological process and a major driver of T cell transcriptional heterogeneity. The EDS is a weighted list of genes that can be used to compute a numerical score per cell, independent of the composition of the dataset, and that we believe provides an scRNA-seq analog to marker-based gating. We also identified a gene module that correlates with T cell memory differentiation (S100A genes) and identified the pattern of granzyme expression as a robust signature of T and NK cell differentiation. The modules presented here are by no means comprehensive, and we hope that the field will build a consensus around validated, standardized, transcript-based measurements of lineage and functional states.

Other analysis strategies are being developed to address similar concerns. The primary alternative approach involves projection of transcriptomes into an established annotated reference dataset, allowing label transfer between a highly annotated reference dataset and incoming data.[9,10] A second related strategy is the use of reference datasets to train machine-learning classifiers that can be used to classify new data. There is merit

to these approaches, and they are not mutually exclusive with the approaches suggested in this study. The challenge with reference-based approaches is that they are extremely dependent on the suitability of the reference atlas to the biological space and the quality of annotation/interpretation of that atlas. We demonstrate that differentiating between cell types that are mutually exclusive (e.g., B cells and T cells) is a very different problem from characterizing finer distinctions within a heterogeneous population with fluid differentiation states (e.g., within T cells). In the latter case, it might not be appropriate to assign cells to mutually exclusive labels. For example, a given cell could simultaneously be cytotoxic, express signatures of exhaustion, and have varying degrees of memory differentiation. In these cases, directly scoring known aspects of biology can yield more useful and interpretable information.

Collectively, we provide the first reference atlas for RM immune cells. We use these data to provide RNA-centric marker definitions of major immune subsets and present a biologically grounded strategy to characterize T cell lineages by modeling key differentiation and functional properties. This is well suited to the biology of immune cells, and the incorporation of this approach will increase the interoperability and interpretability of scRNA-seq data.

Limitations of the study

One limitation of the data presented here is that most samples are from clinically healthy subjects. Thus, many cellular states highly relevant for the study of the immune system are absent or rare in these data. Future work should expand upon this dataset and seek to include inflammatory and activated immune subsets. While RMs are the most common non-human primate model in biomedical research, other species are used, and even closely related species may have evolved differences to some immune subsets. Future work should examine the immune cell transcriptomes of other non-human primate species.

RESOURCE AVAILABILITY

Lead contact

Requests for further information and resources should be directed to and will be fulfilled by the lead contact, Dr. Benjamin Bimber (bimber@ohsu.edu).

Materials availability

This study did not generate new unique reagents.

Data and code availability

The accession numbers for all sequencing data discussed in this manuscript are available in Table S9. The cell classification models developed in this manuscript are part of the RIRA R package, available at https://github.com/bimberlab/RIRA. Gene expression data have been uploaded to the NIH Gene Expression Omnibus (GEO) database, accession GEO: GSE277821, and NIH BioProject: PRJNA1163395.

ACKNOWLEDGMENTS

This work was supported by the National Institute of Allergy and Infectious Diseases (NIAID) grants and contracts 75N93019C00070 (to L.J.P.), P01AI177688-01 (to L.J.P.), and R01AI161010 (to R.K.R.), as well as Bill and Melinda Gates Foundation grant INV-002377 (to L.J.P.) and the Oregon National Primate Research Center Core grant from the NIH, Office of the Director (P51OD011092). The research reported in this publication used computational infrastructure supported by the Office of Research Infrastructure Programs, Office of the Director, of the NIH under award number S10OD034224. The content is solely the responsibility of the authors and does not necessarily represent the official views of the NIH. We thank Dr. Katinka Vigh-Conrad for assistance with figure preparation.

AUTHOR CONTRIBUTIONS

S.O., S.F., B.V.-M. W.G., C.N., D.D., A.S., T.B., A.B.-A., J.V.S., C.S.L., M.K.A., and S.G.H. conducted the underlying animal experiments, performed sample collection and processing, and generated and analyzed immunologic data. E.M., G.J.B., G.W.M., M.Z., S.B., and B.N.B. performed data analysis. B.N.B. conceptualized the project and drafted the manuscript. L.J.P., R.K.R., and A.A.O. secured funding. All authors contributed to manuscript editing.

DECLARATION OF INTERESTS

The authors declare no competing interests.

STAR★METHODS

Detailed methods are provided in the online version of this paper and include the following:

- KEY RESOURCES TABLE
- METHOD DETAILS
  - Animal subjects
  - Tissue collection and processing
  - Flow cytometry and FACS sorting
  - CITE-seq staining and Cell hashing
  - Single-cell RNA sequencing
  - Single-cell RNA-seq pre-processing
  - TCR sequence analysis
  - Data processing for dot plots
  - Human scRNA-seq data

SUPPLEMENTAL INFORMATION

Supplemental information can be found online at https://doi.org/10.1016/j.xgen.2025.100849.

Received: October 8, 2024
Revised: February 17, 2025
Accepted: March 18, 2025
Published: April 14, 2025

REFERENCES

1. Murphy, K., and Weaver, C. (2016). Janeway's Immunobiology, 9th edition (Garland Science/Taylor & Francis Group, LLC).
2. Marsh-Wakefield, F.M., Mitchell, A.J., Norton, S.E., Ashhurst, T.M., Leman, J.K., Roberts, J.M., Harte, J.E., McGuire, H.M., and Kemp, R.A. (2021). Making the most of high-dimensional cytometry data. Immunol. Cell Biol. *99*, 680–696. https://doi.org/10.1111/imcb.12456.
3. Arad, G., and Geiger, T. (2023). Functional Impact of Protein-RNA Variation in Clinical Cancer Analyses. Mol. Cell. Proteomics *22*, 100587. https://doi.org/10.1016/j.mcpro.2023.100587.
4. Gry, M., Rimini, R., Strömberg, S., Asplund, A., Pontén, F., Uhlén, M., and Nilsson, P. (2009). Correlations between RNA and protein expression profiles in 23 human cell lines. BMC Genom. *10*, 365. https://doi.org/10.1186/1471-2164-10-365.
5. Mullan, K.A., de Vrij, N., Valkiers, S., and Meysman, P. (2023). Current annotation strategies for T cell phenotyping of single-cell RNA-seq data. Front. Immunol. *14*, 1306169. https://doi.org/10.3389/fimmu.2023.1306169.

6. Butler, A., Hoffman, P., Smibert, P., Papalexi, E., and Satija, R. (2018). Integrating single-cell transcriptomic data across different conditions, technologies, and species. Nat. Biotechnol. *36*, 411–420. https://doi.org/10.1038/nbt.4096.

7. Sturm, G., Szabo, T., Fotakis, G., Haider, M., Rieder, D., Trajanoski, Z., and Finotello, F. (2020). Scirpy: a Scanpy extension for analyzing single-cell T-cell receptor-sequencing data. Bioinformatics *36*, 4817–4818. https://doi.org/10.1093/bioinformatics/btaa611.

8. Wolf, F.A., Angerer, P., and Theis, F.J. (2018). SCANPY: large-scale single-cell gene expression data analysis. Genome Biol. *19*, 15. https://doi.org/10.1186/s13059-017-1382-0.

9. Lotfollahi, M., Yuhan, H., Theis, F.J., and Satija, R. (2024). The future of rapid and automated single-cell data analysis using reference mapping. Cell *187*, 2343–2358. https://doi.org/10.1016/j.cell.2024.03.009.

10. Andreatta, M., Corria-Osorio, J., Müller, S., Cubas, R., Coukos, G., and Carmona, S.J. (2021). Interpretation of T cell states from single-cell transcriptomics data using reference atlases. Nat. Commun. *12*, 2965. https://doi.org/10.1038/s41467-021-23324-4.

11. Geginat, J., Paroni, M., Maglie, S., Alfen, J.S., Kastirr, I., Gruarin, P., De Simone, M., Pagani, M., and Abrignani, S. (2014). Plasticity of human CD4 T cell subsets. Front. Immunol. *5*, 630. https://doi.org/10.3389/fimmu.2014.00630.

12. Murphy, K.M., and Stockinger, B. (2010). Effector T cell plasticity: flexibility in the face of changing circumstances. Nat. Immunol. *11*, 674–680. https://doi.org/10.1038/ni.1899.

13. Fonseca, R., Beura, L.K., Quarnstrom, C.F., Ghoneim, H.E., Fan, Y., Zebley, C.C., Scott, M.C., Fares-Frederickson, N.J., Wijeyesinghe, S., Thompson, E.A., et al. (2020). Developmental plasticity allows outside-in immune responses by resident memory T cells. Nat. Immunol. *21*, 412–421. https://doi.org/10.1038/s41590-020-0607-7.

14. Omilusik, K.D., and Goldrath, A.W. (2019). Remembering to remember: T cell memory maintenance and plasticity. Curr. Opin. Immunol. *58*, 89–97. https://doi.org/10.1016/j.coi.2019.04.009.

15. Andreatta, M., Berenstein, A.J., and Carmona, S.J. (2022). scGate: marker-based purification of cell types from heterogeneous single-cell RNA-seq datasets. Bioinformatics *38*, 2642–2644. https://doi.org/10.1093/bioinformatics/btac141.

16. Dominguez Conde, C., Xu, C., Jarvis, L.B., Rainbow, D.B., Wells, S.B., Gomes, T., Howlett, S.K., Suchanek, O., Polanski, K., King, H.W., et al. (2022). Cross-tissue immune cell analysis reveals tissue-specific features in humans. Science *376*, eabl5197. https://doi.org/10.1126/science.abl5197.

17. Wight, T.N., Kang, I., Evanko, S.P., Harten, I.A., Chang, M.Y., Pearce, O.M.T., Allen, C.E., and Frevert, C.W. (2020). Versican-A Critical Extracellular Matrix Regulator of Immunity and Inflammation. Front. Immunol. *11*, 512. https://doi.org/10.3389/fimmu.2020.00512.

18. Mei, S., Alchahin, A.M., Embaie, B.T., Gavriliuc, I.M., Verhoeven, B.M., Zhao, T., Li, X., Jeffries, N.E., Pepich, A., Sarkar, H., et al. (2024). Single-cell analyses of metastatic bone marrow in human neuroblastoma reveals microenvironmental remodeling and metastatic signature. JCI Insight *9*, e173337. https://doi.org/10.1172/jci.insight.173337.

19. Jin, R., Hao, J., Yi, Y., Sauter, E., and Li, B. (2021). Regulation of macrophage functions by FABP-mediated inflammatory and metabolic pathways. Biochim. Biophys. Acta. Mol. Cell Biol. Lipids *1866*, 158964. https://doi.org/10.1016/j.bbalip.2021.158964.

20. Han, W., Tanjore, H., Liu, Y., Hunt, R.P., Gutor, S.S., Serezani, A.P.M., and Blackwell, T.S. (2023). Identification and Characterization of Alveolar and Recruited Lung Macrophages during Acute Lung Inflammation. J. Immunol. *210*, 1827–1836. https://doi.org/10.4049/jimmunol.2200694.

21. Chavez-Galan, L., Olleros, M.L., Vesin, D., and Garcia, I. (2015). Much More than M1 and M2 Macrophages, There are also CD169(+) and TCR(+) Macrophages. Front. Immunol. *6*, 263. https://doi.org/10.3389/fimmu.2015.00263.

22. van Dinther, D., Veninga, H., Iborra, S., Borg, E.G.F., Hoogterp, L., Olesek, K., Beijer, M.R., Schetters, S.T.T., Kalay, H., Garcia-Vallejo, J.J., et al. (2018). Functional CD169 on Macrophages Mediates Interaction with Dendritic Cells for CD8(+) T Cell Cross-Priming. Cell Rep. *22*, 1484–1495. https://doi.org/10.1016/j.celrep.2018.01.021.

23. Liu, Y., Xia, Y., and Qiu, C.H. (2021). Functions of CD169 positive macrophages in human diseases. Biomed. Rep. *14*, 26, (Review). https://doi.org/10.3892/br.2020.1402.

24. Musumeci, A., Lutz, K., Winheim, E., and Krug, A.B. (2019). What Makes a pDC: Recent Advances in Understanding Plasmacytoid DC Development and Heterogeneity. Front. Immunol. *10*, 1222. https://doi.org/10.3389/fimmu.2019.01222.

25. Zengin, H.B., Pukhalskaya, T., and Smoller, B.R. (2021). Role of CD123 (+) Plasmacytoid Dendritic Cells in Etiologically Different Variants of Erythema Multiforme: A Monocentric Retrospective Study. Dermatopathology *8*, 89–96. https://doi.org/10.3390/dermatopathology8020014.

26. Kvedaraite, E., and Ginhoux, F. (2022). Human dendritic cells in cancer. Sci. Immunol. *7*, eabm9409. https://doi.org/10.1126/sciimmunol.abm9409.

27. Zhang, Q., He, Y., Luo, N., Patel, S.J., Han, Y., Gao, R., Modak, M., Carotta, S., Haslinger, C., Kind, D., et al. (2019). Landscape and Dynamics of Single Immune Cells in Hepatocellular Carcinoma. Cell *179*, 829–845.e20. https://doi.org/10.1016/j.cell.2019.10.003.

28. Zilionis, R., Engblom, C., Pfirschke, C., Savova, V., Zemmour, D., Saatcioglu, H.D., Krishnan, I., Maroni, G., Meyerovitz, C.V., Kerwin, C.M., et al. (2019). Single-Cell Transcriptomics of Human and Mouse Lung Cancers Reveals Conserved Myeloid Populations across Individuals and Species. Immunity *50*, 1317–1334.e10. https://doi.org/10.1016/j.immuni.2019.03.009.

29. Maier, B., Leader, A.M., Chen, S.T., Tung, N., Chang, C., LeBerichel, J., Chudnovskiy, A., Maskey, S., Walker, L., Finnigan, J.P., et al. (2020). Author Correction: A conserved dendritic-cell regulatory program limits antitumour immunity. Nature *582*, E17. https://doi.org/10.1038/s41586-020-2326-5.

30. Yamawaki, T.M., Lu, D.R., Ellwanger, D.C., Bhatt, D., Manzanillo, P., Arias, V., Zhou, H., Yoon, O.K., Homann, O., Wang, S., and Li, C.M. (2021). Systematic comparison of high-throughput single-cell RNA-seq methods for immune cell profiling. BMC Genom. *22*, 66. https://doi.org/10.1186/s12864-020-07358-4.

31. Kapellos, T.S., Baßler, K., Fujii, W., Nalkurthi, C., Schaar, A.C., Bonaguro, L., Pecht, T., Galvao, I., Agrawal, S., Saglam, A., et al. (2023). Systemic alterations in neutrophils and their precursors in early-stage chronic obstructive pulmonary disease. Cell Rep. *42*, 112525. https://doi.org/10.1016/j.celrep.2023.112525.

32. Olsson, A., Venkatasubramanian, M., Chaudhri, V.K., Aronow, B.J., Salomonis, N., Singh, H., and Grimes, H.L. (2016). Single-cell analysis of mixed-lineage states leading to a binary cell fate choice. Nature *537*, 698–702. https://doi.org/10.1038/nature19348.

33. Iwakoshi, N.N., Lee, A.H., Vallabhajosyula, P., Otipoby, K.L., Rajewsky, K., and Glimcher, L.H. (2003). Plasma cell differentiation and the unfolded protein response intersect at the transcription factor XBP-1. Nat. Immunol. *4*, 321–329. https://doi.org/10.1038/ni907.

34. Shi, G.X., Harrison, K., Wilson, G.L., Moratz, C., and Kehrl, J.H. (2002). RGS13 regulates germinal center B lymphocytes responsiveness to CXC chemokine ligand (CXCL)12 and CXCL13. J. Immunol. *169*, 2507–2515. https://doi.org/10.4049/jimmunol.169.5.2507.

35. Brescia, P., Schneider, C., Holmes, A.B., Shen, Q., Hussein, S., Pasqualucci, L., Basso, K., and Dalla-Favera, R. (2018). MEF2B Instructs Germinal Center Development and Acts as an Oncogene in B Cell Lymphomagenesis. Cancer Cell *34*, 453–465.e9. https://doi.org/10.1016/j.ccell.2018.08.006.

36. Boulianne, B., Le, M.X., Ward, L.A., Meng, L., Haddad, D., Li, C., Martin, A., and Gommerman, J.L. (2013). AID-expressing germinal center B cells cluster normally within lymph node follicles in the absence of FDC-M1+

CD35+ follicular dendritic cells but dissipate prematurely. J. Immunol. *191*, 4521–4530. https://doi.org/10.4049/jimmunol.1300769.

37. Valentin Hansen, S., Høy Hansen, M., Cédile, O., Møller, M.B., Haaber, J., Abildgaard, N., and Guldborg Nyvold, C. (2021). Detailed characterization of the transcriptome of single B cells in mantle cell lymphoma suggesting a potential use for SOX4. Sci. Rep. *11*, 19092. https://doi.org/10.1038/s41598-021-98560-1.

38. Bautista, D., Vásquez, C., Ayala-Ramírez, P., Téllez-Sosa, J., Godoy-Lozano, E., Martínez-Barnetche, J., Franco, M., and Angel, J. (2020). Differential Expression of IgM and IgD Discriminates Two Subpopulations of Human Circulating IgM(+)IgD(+)CD27(+) B Cells That Differ Phenotypically, Functionally, and Genetically. Front. Immunol. *11*, 736. https://doi.org/10.3389/fimmu.2020.00736.

39. Heng, T.S.P., and Painter, M.W.; Immunological Genome Project Consortium (2008). The Immunological Genome Project: networks of gene expression in immune cells. Nat. Immunol. *9*, 1091–1094. https://doi.org/10.1038/ni1008-1091.

40. Rakhmanov, M., Sic, H., Kienzler, A.K., Fischer, B., Rizzi, M., Seidl, M., Melkaoui, K., Unger, S., Moehle, L., Schmit, N.E., et al. (2014). High levels of SOX5 decrease proliferative capacity of human B cells, but permit plasmablast differentiation. PLoS One *9*, e100328. https://doi.org/10.1371/journal.pone.0100328.

41. Asano, Y., Daccache, J., Jain, D., Ko, K., Kinloch, A., Veselits, M., Wolfgeher, D., Chang, A., Josephson, M., Cunningham, P., et al. (2021). Innate-like self-reactive B cells infiltrate human renal allografts during transplant rejection. Nat. Commun. *12*, 4372. https://doi.org/10.1038/s41467-021-24615-6.

42. Liu, K., Hoover, A.R., Krawic, J.R., DeVette, C.I., Sun, X.H., Hildebrand, W.H., Lang, M.L., Axtell, R.C., and Chen, W.R. (2022). Antigen presentation and interferon signatures in B cells driven by localized ablative cancer immunotherapy correlate with extended survival. Theranostics *12*, 639–656. https://doi.org/10.7150/thno.65773.

43. Laidlaw, B.J., and Cyster, J.G. (2021). Transcriptional regulation of memory B cell differentiation. Nat. Rev. Immunol. *21*, 209–220. https://doi.org/10.1038/s41577-020-00446-2.

44. Luo, Y., Wang, J., Li, K., Li, M., Xu, S., Liu, X., Zhang, Z., Xu, X., Zhang, Y., Pan, J., et al. (2022). Single-cell genomics identifies distinct B1 cell developmental pathways and reveals aging-related changes in the B-cell receptor repertoire. Cell Biosci. *12*, 57. https://doi.org/10.1186/s13578-022-00795-6.

45. Laidlaw, B.J., Duan, L., Xu, Y., Vazquez, S.E., and Cyster, J.G. (2020). The transcription factor Hhex cooperates with the corepressor Tle3 to promote memory B cell development. Nat. Immunol. *21*, 1082–1093. https://doi.org/10.1038/s41590-020-0713-6.

46. Kim, W., Zhou, J.Q., Horvath, S.C., Schmitz, A.J., Sturtz, A.J., Lei, T., Liu, Z., Kalaidina, E., Thapa, M., Alsoussi, W.B., et al. (2022). Germinal centre-driven maturation of B cell response to mRNA vaccination. Nature *604*, 141–145. https://doi.org/10.1038/s41586-022-04527-1.

47. Seifert, M., Sellmann, L., Bloehdorn, J., Wein, F., Stilgenbauer, S., Dürig, J., and Küppers, R. (2012). Cellular origin and pathophysiology of chronic lymphocytic leukemia. J. Exp. Med. *209*, 2183–2198. https://doi.org/10.1084/jem.20120833.

48. Neumann, B., Klippert, A., Raue, K., Sopper, S., and Stahl-Hennig, C. (2015). Characterization of B and plasma cells in blood, bone marrow, and secondary lymphoid organs of rhesus macaques by multicolor flow cytometry. J. Leukoc. Biol. *97*, 19–30. https://doi.org/10.1189/jlb.1HI0514-243R.

49. Andreatta, M., and Carmona, S.J. (2021). UCell: Robust and scalable single-cell gene signature scoring. Comput. Struct. Biotechnol. J. *19*, 3796–3798. https://doi.org/10.1016/j.csbj.2021.06.043.

50. Aran, D., Looney, A.P., Liu, L., Wu, E., Fong, V., Hsu, A., Chak, S., Naikawadi, R.P., Wolters, P.J., Abate, A.R., et al. (2019). Reference-based analysis of lung single-cell sequencing reveals a transitional profibrotic macrophage. Nat. Immunol. *20*, 163–172. https://doi.org/10.1038/s41590-018-0276-y.

51. Gardiner, C.M. (2015). NK cell function and receptor diversity in the context of HCV infection. Front. Microbiol. *6*, 1061. https://doi.org/10.3389/fmicb.2015.01061.

52. Alcover, A., Alarcón, B., and Di Bartolo, V. (2018). Cell Biology of T Cell Receptor Expression and Regulation. Annu. Rev. Immunol. *36*, 103–125. https://doi.org/10.1146/annurev-immunol-042617-053429.

53. Antonacci, R., Massari, S., Linguiti, G., Caputi Jambrenghi, A., Giannico, F., Lefranc, M.P., and Ciccarese, S. (2020). Evolution of the T-Cell Receptor (TR) Loci in the Adaptive Immune Response: The Tale of the TRG Locus in Mammals. Genes *11*, 624. https://doi.org/10.3390/genes11060624.

54. Hayday, A.C., and Pennington, D.J. (2007). Key factors in the organized chaos of early T cell development. Nat. Immunol. *8*, 137–144. https://doi.org/10.1038/ni1436.

55. Cenerenti, M., Saillard, M., Romero, P., and Jandus, C. (2022). The Era of Cytotoxic CD4 T Cells. Front. Immunol. *13*, 867189. https://doi.org/10.3389/fimmu.2022.867189.

56. Kaech, S.M., and Ahmed, R. (2001). Memory CD8+ T cell differentiation: initial antigen encounter triggers a developmental program in naive cells. Nat. Immunol. *2*, 415–422. https://doi.org/10.1038/87720.

57. Pitcher, C.J., Hagen, S.I., Walker, J.M., Lum, R., Mitchell, B.L., Maino, V.C., Axthelm, M.K., and Picker, L.J. (2002). Development and homeostasis of T cell memory in rhesus macaque. J. Immunol. *168*, 29–43. https://doi.org/10.4049/jimmunol.168.1.29.

58. Butcher, E.C., and Picker, L.J. (1996). Lymphocyte homing and homeostasis. Science *272*, 60–66. https://doi.org/10.1126/science.272.5258.60.

59. Weatherly, K., Bettonville, M., Torres, D., Kohler, A., Goriely, S., and Braun, M.Y. (2015). Functional profile of S100A4-deficient T cells. Immun. Inflamm. Dis. *3*, 431–444. https://doi.org/10.1002/iid3.85.

60. Neefjes, J., Jongsma, M.L.M., Paul, P., and Bakke, O. (2011). Towards a systems understanding of MHC class I and MHC class II antigen presentation. Nat. Rev. Immunol. *11*, 823–836. https://doi.org/10.1038/nri3084.

61. Rout, P., Caminero, F., Iqbal, Z., and Tadi, P.. Histology, Cytotoxic T Cells. In StatPearls. https://www.ncbi.nlm.nih.gov/books/NBK559279/.

62. Sobanski, T., Suraweera, A., Burgess, J.T., Richard, I., Cheong, C.M., Dave, K., Rose, M., Adams, M.N., O'Byrne, K.J., Richard, D.J., and Bolderson, E. (2023). The fructose-bisphosphate, Aldolase A (ALDOA), facilitates DNA-PKcs and ATM kinase activity to regulate DNA double-strand break repair. Sci. Rep. *13*, 15171. https://doi.org/10.1038/s41598-023-41133-1.

63. Jurgens, A.P., Popović, B., and Wolkers, M.C. (2021). T cells at work: How post-transcriptional mechanisms control T cell homeostasis and activation. Eur. J. Immunol. *51*, 2178–2187. https://doi.org/10.1002/eji.202049055.

64. Zhu, Z., Lou, G., Teng, X.L., Wang, H., Luo, Y., Shi, W., Yihunie, K., Hao, S., DeGolier, K., Liao, C., et al. (2024). FOXP1 and KLF2 reciprocally regulate checkpoints of stem-like to effector transition in CAR T cells. Nat. Immunol. *25*, 117–128. https://doi.org/10.1038/s41590-023-01685-w.

65. Zhang, B., Upadhyay, R., Hao, Y., Samanovic, M.I., Herati, R.S., Blair, J.D., Axelrad, J., Mulligan, M.J., Littman, D.R., and Satija, R. (2023). Multimodal single-cell datasets characterize antigen-specific CD8(+) T cells across SARS-CoV-2 vaccination and infection. Nat. Immunol. *24*, 1725–1734. https://doi.org/10.1038/s41590-023-01608-9.

66. Carville, A., Evans, T.I., and Reeves, R.K. (2013). Characterization of circulating natural killer cells in neotropical primates. PLoS One *8*, e78793. https://doi.org/10.1371/journal.pone.0078793.

67. Kabelitz, D., Kalyan, S., Oberg, H.H., and Wesch, D. (2013). Human Vdelta2 versus non-Vdelta2 gammadelta T cells in antitumor immunity. OncoImmunology *2*, e23304. https://doi.org/10.4161/onci.23304.

68. Osinska, I., Popko, K., and Demkow, U. (2014). Perforin: an important player in immune response. Cent. Eur. J. Immunol. *39*, 109–115. https://doi.org/10.5114/ceji.2014.42135.

69. Pipkin, M.E., Rao, A., and Lichtenheld, M.G. (2010). The transcriptional control of the perforin locus. Immunol. Rev. *235*, 55–72. https://doi.org/10.1111/j.0105-2896.2010.00905.x.

70. Tewary, P., Yang, D., de la Rosa, G., Li, Y., Finn, M.W., Krensky, A.M., Clayberger, C., and Oppenheim, J.J. (2010). Granulysin activates antigen-presenting cells through TLR4 and acts as an immune alarmin. Blood *116*, 3465–3474. https://doi.org/10.1182/blood-2010-03-273953.

71. Pankow, A., and Sun, X.H. (2022). The divergence between T cell and innate lymphoid cell fates controlled by E and Id proteins. Front. Immunol. *13*, 960444. https://doi.org/10.3389/fimmu.2022.960444.

72. Ghosh, A., Khanam, A., Ray, K., Mathur, P., Subramanian, A., Poonia, B., and Kottilil, S. (2023). CD38: an ecto-enzyme with functional diversity in T cells. Front. Immunol. *14*, 1146791. https://doi.org/10.3389/fimmu.2023.1146791.

73. Kumar, B.V., Ma, W., Miron, M., Granot, T., Guyer, R.S., Carpenter, D.J., Senda, T., Sun, X., Ho, S.H., Lerner, H., et al. (2017). Human Tissue-Resident Memory T Cells Are Defined by Core Transcriptional and Functional Signatures in Lymphoid and Mucosal Sites. Cell Rep. *20*, 2921–2934. https://doi.org/10.1016/j.celrep.2017.08.078.

74. Bromley, S.K., Akbaba, H., Mani, V., Mora-Buch, R., Chasse, A.Y., Sama, A., and Luster, A.D. (2020). CD49a Regulates Cutaneous Resident Memory CD8(+) T Cell Persistence and Response. Cell Rep. *32*, 108085. https://doi.org/10.1016/j.celrep.2020.108085.

75. Walzer, T., Marçais, A., Saltel, F., Bella, C., Jurdic, P., and Marvel, J. (2003). Cutting edge: immediate RANTES secretion by resting memory CD8 T cells following antigenic stimulation. J. Immunol. *170*, 1615–1619. https://doi.org/10.4049/jimmunol.170.4.1615.

76. Li, C., Phoon, Y.P., Karlinsey, K., Tian, Y.F., Thapaliya, S., Thongkum, A., Qu, L., Matz, A.J., Cameron, M., Cameron, C., et al. (2022). A high OXPHOS CD8 T cell subset is predictive of immunotherapy resistance in melanoma patients. J. Exp. Med. *219*, e20202084. https://doi.org/10.1084/jem.20202084.

77. Li, F., Liu, H., Zhang, D., Ma, Y., and Zhu, B. (2022). Metabolic plasticity and regulation of T cell exhaustion. Immunology *167*, 482–494. https://doi.org/10.1111/imm.13575.

78. Wiseman, R.W., Karl, J.A., Bimber, B.N., O'Leary, C.E., Lank, S.M., Tuscher, J.J., Detmer, A.M., Bouffard, P., Levenkova, N., Turcotte, C.L., et al. (2009). Major histocompatibility complex genotyping with massively parallel pyrosequencing. Nat. Med. *15*, 1322–1326. https://doi.org/10.1038/nm.2038.

79. Dudley, D.M., Karl, J.A., Creager, H.M., Bohn, P.S., Wiseman, R.W., and O'Connor, D.H. (2014). Full-length novel MHC class I allele discovery by next-generation sequencing: two platforms are better than one. Immunogenetics *66*, 15–24. https://doi.org/10.1007/s00251-013-0744-3.

80. Zevin, A.S., Moats, C., May, D., Wangari, S., Miller, C., Ahrens, J., Iwayama, N., Brown, M., Bratt, D., Klatt, N.R., and Smedley, J. (2017). Laparoscopic Technique for Serial Collection of Liver and Mesenteric Lymph Nodes in Macaques. J. Vis. Exp. 55617. https://doi.org/10.3791/55617.

81. Kauffman, K.D., Sallin, M.A., Sakai, S., Kamenyeva, O., Kabat, J., Weiner, D., Sutphin, M., Schimel, D., Via, L., Barry, C.E., 3rd., et al. (2018). Defective positioning in granulomas but not lung-homing limits CD4 T-cell interactions with Mycobacterium tuberculosis-infected macrophages in rhesus macaques. Mucosal Immunol. *11*, 462–473. https://doi.org/10.1038/mi.2017.60.

82. Burwitz, B.J., Wettengel, J.M., Mück-Häusl, M.A., Ringelhan, M., Ko, C., Festag, M.M., Hammond, K.B., Northrup, M., Bimber, B.N., Jacob, T., et al. (2017). Hepatocytic expression of human sodium-taurocholate co-transporting polypeptide enables hepatitis B virus infection of macaques. Nat. Commun. *8*, 2146. https://doi.org/10.1038/s41467-017-01953-y.

83. McGinnis, C.S., Patterson, D.M., Winkler, J., Conrad, D.N., Hein, M.Y., Srivastava, V., Hu, J.L., Murrow, L.M., Weissman, J.S., Werb, Z., et al. (2019). MULTI-seq: sample multiplexing for single-cell RNA sequencing using lipid-tagged indices. Nat. Methods *16*, 619–626. https://doi.org/10.1038/s41592-019-0433-8.

84. Xin, H., Lian, Q., Jiang, Y., Luo, J., Wang, X., Erb, C., Xu, Z., Zhang, X., Heidrich-O'Hare, E., Yan, Q., et al. (2020). GMM-Demux: sample demultiplexing, multiplet detection, experiment planning, and novel cell-type verification in single cell sequencing. Genome Biol. *21*, 188. https://doi.org/10.1186/s13059-020-02084-2.

85. Gaublomme, J.T., Li, B., McCabe, C., Knecht, A., Yang, Y., Drokhlyansky, E., Van Wittenberghe, N., Waldman, J., Dionne, D., Nguyen, L., et al. (2019). Nuclei multiplexing with barcoded antibodies for single-nucleus genomics. Nat. Commun. *10*, 2907. https://doi.org/10.1038/s41467-019-10756-2.

86. Boggy, G.J., McElfresh, G.W., Mahyari, E., Ventura, A.B., Hansen, S.G., Picker, L.J., and Bimber, B.N. (2022). BFF and cellhashR: Analysis Tools for Accurate Demultiplexing of Cell Hashing Data. Bioinformatics *38*, 2791–2801. https://doi.org/10.1093/bioinformatics/btac213.

87. McGinnis, C.S., Murrow, L.M., and Gartner, Z.J. (2019). DoubletFinder: Doublet Detection in Single-Cell RNA Sequencing Data Using Artificial Nearest Neighbors. Cell Syst. *8*, 329–337.e4. https://doi.org/10.1016/j.cels.2019.03.003.

STAR★METHODS

KEY RESOURCES TABLE

REAGENT or RESOURCE	SOURCE	IDENTIFIER
Antibodies		
CITE-seq TotalSeq-C Antibodies	BioLegend	Antibodies **and** concentrations listed in Table S1
Biological samples		
Tissues from rhesus macaques were collected as described in the STAR Methods		N/A
Critical commercial assays		
10x Genomics 5′ V2/HT Reagents	10x Genomics	N/A
Deposited data		
Raw sequence data have been deposited in the NIH Short Read Archive (SRA) database	NIH SRA DB	Full listing of SRA accessions provided in Table S9
Processed scRNA-seq expression data are available through the NIH GEO database	NIH GEO DB	GEO Accession: GSE277821
Oligonucleotides		
Oligos for TCR amplification listed in Table S6		N/A
Software and algorithms		
Methods generated in this manuscript have been incorporated into the RIRA R package (https://github.com/BimberLab/RIRA)	GitHub	https://doi.org/10.5281/zenodo.14976334

METHOD DETAILS

Animal subjects

The RIRA cohort comprises 47 Rhesus macaques (*Macaca mulatta*) primarily of Indian genetic background, with two Indian/Chinese hybrid animals. Table S1 contains a summary of demographic information for these samples. All study macaques were housed at the Oregon National Primate Research Center (ONPRC) in animal biosafety level 2 rooms with autonomously controlled temperature, humidity, and lighting. Macaques were fed commercially prepared primate chow twice daily and received supplemental fresh fruit or vegetables daily. Fresh, potable water was provided via automatic water systems. During all protocol time points, body weight and complete blood counts were collected and animals underwent anesthesia support and monitoring. The ONPRC Institutional Animal Care and Use Committee approved macaque care and all experimental protocols and procedures. The ONPRC is a Category I facility. The American Association for Accreditation of Laboratory Animal Care fully accredits the Laboratory Animal Care and Use Program at the ONPRC. It has an approved assurance (no. A3304-01) for the care and use of animals on file with the National Institutes of Health Office for Protection from Research Risks. The Institutional Animal Care and Use Committee adheres to national guidelines established in the Animal Welfare Act (7 U.S. Code, sections 2131–2159) and the Guide for the Care and Use of Laboratory Animals, Eighth Edition, as mandated by the U.S. Public Health Service Policy. All subsets were naturally infected with wild-type cytomegalovirus at a young age. Genotyping for Major Histocompatibility Complex class I (MHC-I) allele was performed, as previously described.[78,79]

Tissue collection and processing

Cell isolation from PBMC and solid tissues were acquired and processed to single-cell suspensions using previously published methods, summarized below.[5,19] Liver, spleen, and mesenteric lymph node biopsies were collected by a minimally invasive laparoscopic procedure.[80] For lung samples, animals were humanely euthanized, and caudal lung lobe samples were collected during necropsy. Bone marrow cells were harvested from the humerus or iliac crest by flushing with R10 media. Peripheral blood mononuclear cells (PBMCs) were isolated from freshly collected ACD-A treated blood utilizing Ficoll-Paque density centrifugation.[20] Lymph nodes (LN) and spleen samples were homogenized as previously described[57] while liver and lung samples were enzymatically digested with DNAse and collagenase.[81,82] Prior to processing, cells were filtered using 70 μm strainers. Cells were quantified using a Countess II (Thermo Fisher), aliquoted, diluted as required for single-cell RNA sequencing (typically 500-1,500 cells/uL), and kept on ice prior to processing.

Flow cytometry and FACS sorting

PBMC were isolated from anticoagulant-treated whole blood by Ficoll density gradient centrifugation (GE Healthcare). Cells were stained for anti-CD3 (clone: SP34-2, Pacific Blue, BD Biosciences, RRID:AB_397044), anti-CD8 (clone: SK1, PerCP-eFluor710, BD Biosciences, RRID:AB_400095), anti-CD4 (clone: L200, PE-Cy7, BD Biosciences, RRID:AB_1727474), anti-CD95 (clone: DX2, PE, BioLegend, RRID:AB_10896481), anti-CD28 (CD28.2, PE/Dazzle 594, BioLegend, RRID:AB_11151918), anti TCRγδ (clone: B1, FITC, BioLegend, RRID:AB_10895569), and LIVE/DEAD Fixable Near Infra-Red Dead Cell Stain (Life Technologies) and were incubated for an additional 30 min in the dark at 4°C. Cells were then washed once with 1x PBS. Viable cells were sorted using a FACSAria Fusion (BD Biosciences), and analysis was conducted using a combination of FACSDiva software (BD Biosciences) and FlowJo software (BD).

CITE-seq staining and Cell hashing

Cells were stained using a panel of 27 nucleotide-labeled antibodies (BioLegend TotalSeq-C), using the concentrations listed in Table S2, diluted in PBS with 0.5% BSA. To multiplex samples, cell hashing was used, with the MULTI-Seq lipid labeling system,[83] using commercially available lipid modified oligos (Sigma Aldridge LMO001). Concurrent CITE-seq and MultiSeq labeling was performed as follows. Cells were incubated with the CITE-seq antibody cocktail in 30uL total volume for 30 min at 4°C. Next, 15uL MultiSeq solution 1 (LMO stock, diluted in PBS to 400nM) was added, along with 45 μL of the barcode solution (10μM barcode oligo, diluted in PBS to 400nM), giving a final working concentration of 200nM for LMO and 200nM for the barcode oligo. Next, pipet mix and incubate for 5 min at 4°C. Add 10uL of the MultiSeq co-anchor solution (50μM Co-A stock, diluted in PBS to 2uM), then pipet mix and incubate for 5 min at 4°C. Wash twice with 1 mL cold PBS, spinning cells at 700 g for 5 min at 4°C, and then resuspend in 200 μL R10 (which will quench LMOs). Samples were pooled, followed by GEM generation on the 10x instrument.

Single-cell RNA sequencing

The isolated single cell suspensions were then processed for single-cell RNA sequencing using the 10x Genomics Chromium platform, using 5′ v2 or HT chemistry, following the manufacturer's protocols, including feature barcoding library preparation. To improve capture of MULTI-Seq fragments, we added the following primer, 5′-CCTTGGCACCCGAGAATTCC-3′, at 2.5uM to the 10x cDNA synthesis step. Generation of VDJ enriched libraries followed manufacturer's instructions with the exception that macaque-specific TCR constant region primers were used in place of human-specific TCR enrichment primers for macaque cells (Table S6). Primer pairs were used to amplify the alpha, beta, delta, and gamma TCR chains. The concentration of the alpha constant region primer was increased relative to the beta primer to improve amplification. PCR conditions for both reactions were as follows: lid temp 105°C, 98°C 0:45, 12 cycles of: 98°C 0:20, 60°C 0:30, 72°C 1:00, followed by 72°C 1:00°C and 4°C hold. Sequence libraries were sequenced using Illumina chemistry, on either Novaseq or HiSeq instruments (Illumina).

Single-cell RNA-seq pre-processing

Raw sequence reads were processed using 10X Genomics Cell Ranger software (version 6.1.1). The resulting sequence data were aligned to the MMul_10 genome (assembly ID: GCF_003339765.1) with NCBI gene build 103. Cell demultiplexing used a combination of algorithms, including GMM-demux, demuxEM and BFF, implemented using the cellhashR package.[84–86] Droplets identified as doublets (i.e., the collision of distinct sample barcodes) were removed from downstream analyses. We additionally performed doublet detection using DoubletFinder, and removed doublets from downstream analysis.[87] Next, droplets were filtered based on UMI count (allowing 0–20,000 UMIs/cell), and unique features (allowing 200–5000 features/cell). Additionally, we computed a per-cell saturation statistic for both RNA and Antibody-Derived Tag (ADT) data, defined as: 1 – (#UMIs/#Counts). This statistic provides a per-cell measurement of the completeness with which unique molecules are sampled per cell and has the benefit of being adaptable across diverse cell types. Data were filtered to require RNA saturation >0.35 and ADT saturation >0.1. Analyses utilized the Seurat R package, version 4.2.[6] Using standardized methods implemented in the Seurat R package, counts and UMIs were normalized across cells, scaled per 10,000 bases, and converted to log scale using the 'NormalizeData' function. These values were then converted to z-scores using the 'ScaleData' command. Highly variable genes were selected using the 'FindVariableGenes' function with a dispersion cutoff of 0.5. The top variable gene list was filtered to exclude genes that produce inaccurate counts, such as the HLA class I region, where high polymorphism across the species cannot be represented appropriately in a single reference genome, as well as gene sets empirically identified to be prone to sample-to-sample batch effects, such as ribosomal genes (full list in Table S7). These genes are excluded from PCA/UMAP only. Principal components were calculated for the remaining top variable genes and projected onto all other genes using the 'RunPCA' and 'ProjectPCA' commands. Clusters of similar cells were identified using the Louvain method for community detection, and UMAP projections were calculated. CITE-seq data were Centered log ratio (CLR) normalized by lane, meaning raw count data are subset per lane, CLR normalization performed (as implemented in the Seurat R package, using margin = 1), using all QC-passing cells/lane. Normalization per-lane was performed to reduce batch effects. After normalization, per-feature-UCell values were calculated for each tag. To illustrate the results of alternate informatics pipelines, we additionally processed a subset of data using the ScanPy scRNA-seq pipeline (version 1.10.4).[8]

TCR sequence analysis

Raw sequence reads for gene expression and TCR enrichment were first processed using cellranger software, version 6.1.1 (10X Genomics). For TCR analyses, data were aligned using a custom macaque V/J segment library, developed by merging the IMGT library (2021-09-21 release) with V/J segments identified as part of this study (Table S8). This segment library was provided to the cellranger vdj software. The raw clonotype calls produced by cellranger vdj were extracted from the comma-delimited outputs. Cells were demultiplexed and TCR calls were assigned to samples using custom software, made publicly available through the cellhashR package, with the GMM-Demux, demuxEM, and BFF algorithms.[84–86] The clonotype data generated by cellranger were filtered to drop any cells where the TCR calls lacking a CDR3 sequence, the clonotype was not marked as full-length, or the clonotype lacked a called V, J, or constant gene. Rows with chimeric V/J/C combinations (i.e., TRBV/TRAC) were filtered, with the exception that segments consisting of a TRDV/TRAC or TRAV/TRDC were permitted. These chimeric segments were classified according to the constant region chain.

Data processing for dot plots

The cell-type-specific multimodal dot plots shown in multiple figures, contain dot plots for RNA Markers, Surface Protein, and Tissue Enrichment. The color scale for the RNA Markers dot plot shows average expression (following library-size normalization) and the dot size is determined by the fraction of cells expressing markers (as determined by Seurat's DotPlot function). The color scale for the Surface Protein plot shows the average signal after CLR normalization, while the dot size is determined by the fraction of cells labeled as positive. The color scale for the Tissue Enrichment dot plot shows linearly scaled chi-squared test standardized residuals, while dot size is determined by the number of cells of a cell type in a tissue, scaled by the total number of cells in that tissue.

Human scRNA-seq data

We obtained 10X 5′ single cell RNA-seq data from a previously published human COVID-19 dataset.[65] The dataset included transcriptomic data, TCR sequencing, and surface staining using dextramers loaded with peptides of SARS-CoV-2 spike protein. We re-processed the count matrix by performing PCA/UMAP as described above for the RIRA dataset. All cell labels, dextramer count data and TCR CDR3 data were provided in the original publication and used as-is.

Cell Genomics, Volume *5*

Supplemental information

Enhanced interpretation of immune cell phenotype and function through a rhesus macaque single-cell atlas

Eisa Mahyari, Gregory J. Boggy, G.W. McElfresh, Maanasa Kaza, Sebastian Benjamin, Benjamin Varco-Merth, Sohita Ojha, Shana Feltham, William Goodwin, Candice Nkoy, Derick Duell, Andrea Selseth, Tyler Bennett, Aaron Barber-Axthelm, Jeremy V. Smedley, Caralyn S. Labriola, Michael K. Axthelm, R. Keith Reeves, Afam A. Okoye, Scott G. Hansen, Louis J. Picker, and Benjamin N. Bimber

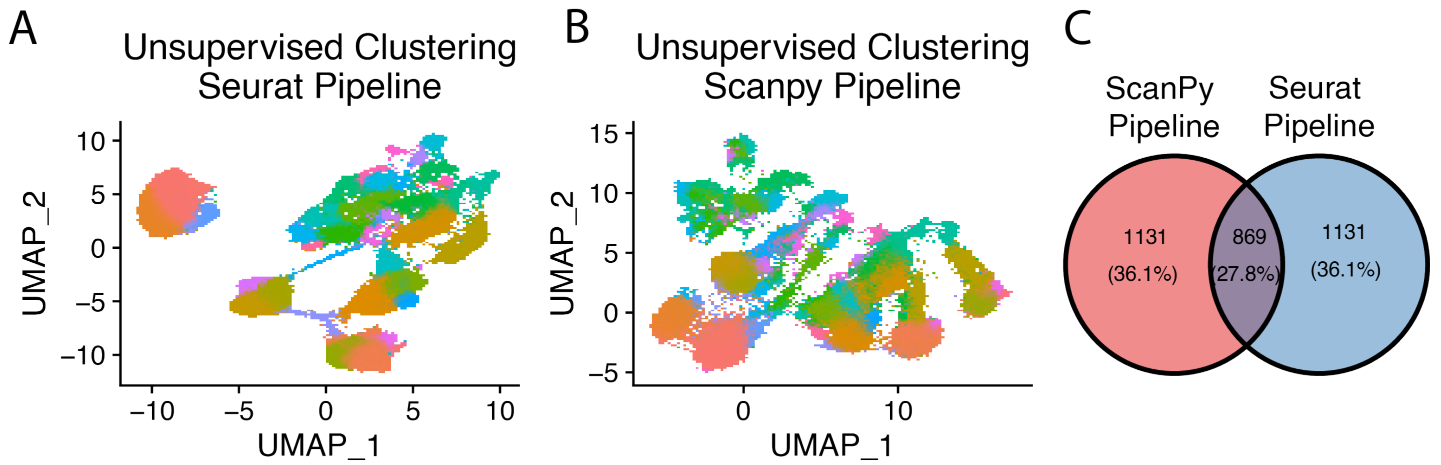

Figure S1. Comparison of dimensionality reduction between Seurat and ScanPy pipelines, related to Figure 5. The data used in these analyses are identical to Figure 5. Reference populations of peripheral T cells were sorted by FACS, producing 56,637 cells, followed by scRNA-seq. A) The data were processed using the pipeline implemented by the Seurat R package, with dimensionality reduction (PCA/UMAP) reference T cells, colored by population. Dimensionality reduction was performed on the top 3,000 variable genes. B) The same input data as (A), with processing and dimensionality reduction performed using the ScanPy python pipeline. Dimensionality reduction was performed on the top 3,000 variable genes. C) The Venn diagram shows the overlap between the top variable genes identified by the Seurat pipeline and the ScanPy pipeline. This difference will influence the outcome of PCA/UMAP and unsupervised clustering.

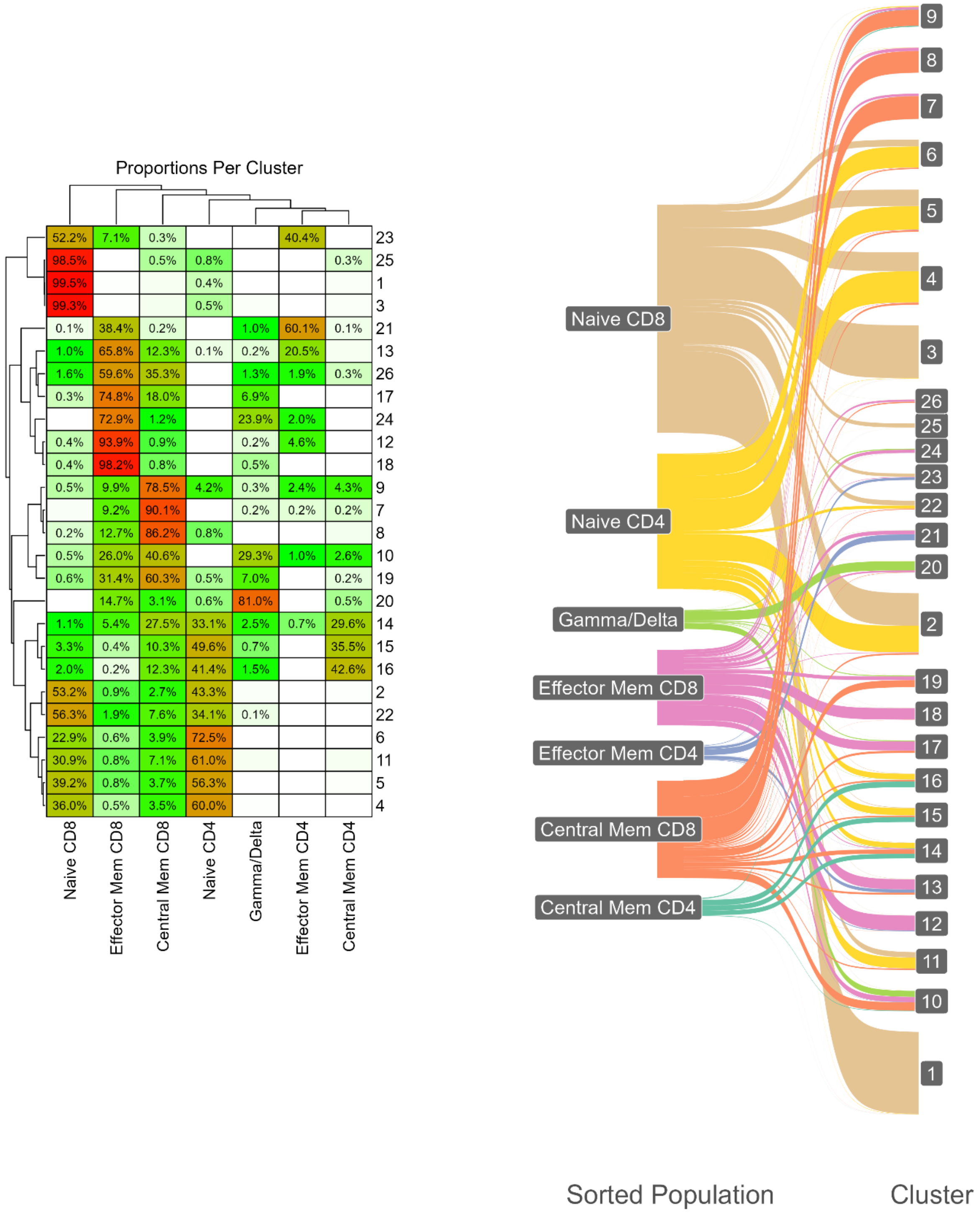

Figures S2. Comparison of unsupervised clustering using different scRNA-seq pipelines and algorithms, related to Figure 5. We performed unsupervised clustering on the data from Figure S1B (ScanPy pipeline) using the leiden clustering algorithm. The heatmap (left) and Sankey diagram (right) contrast the ground-truth population (defined by the cell sort) relative to the cluster assignment. While specific clusters can be generated that are pure or nearly pure for a single population, most clusters are a mixture of reference populations. These data demonstrate that the factors that drive the unsupervised clustering of T cells are not inherently aligned with established lineage definitions.

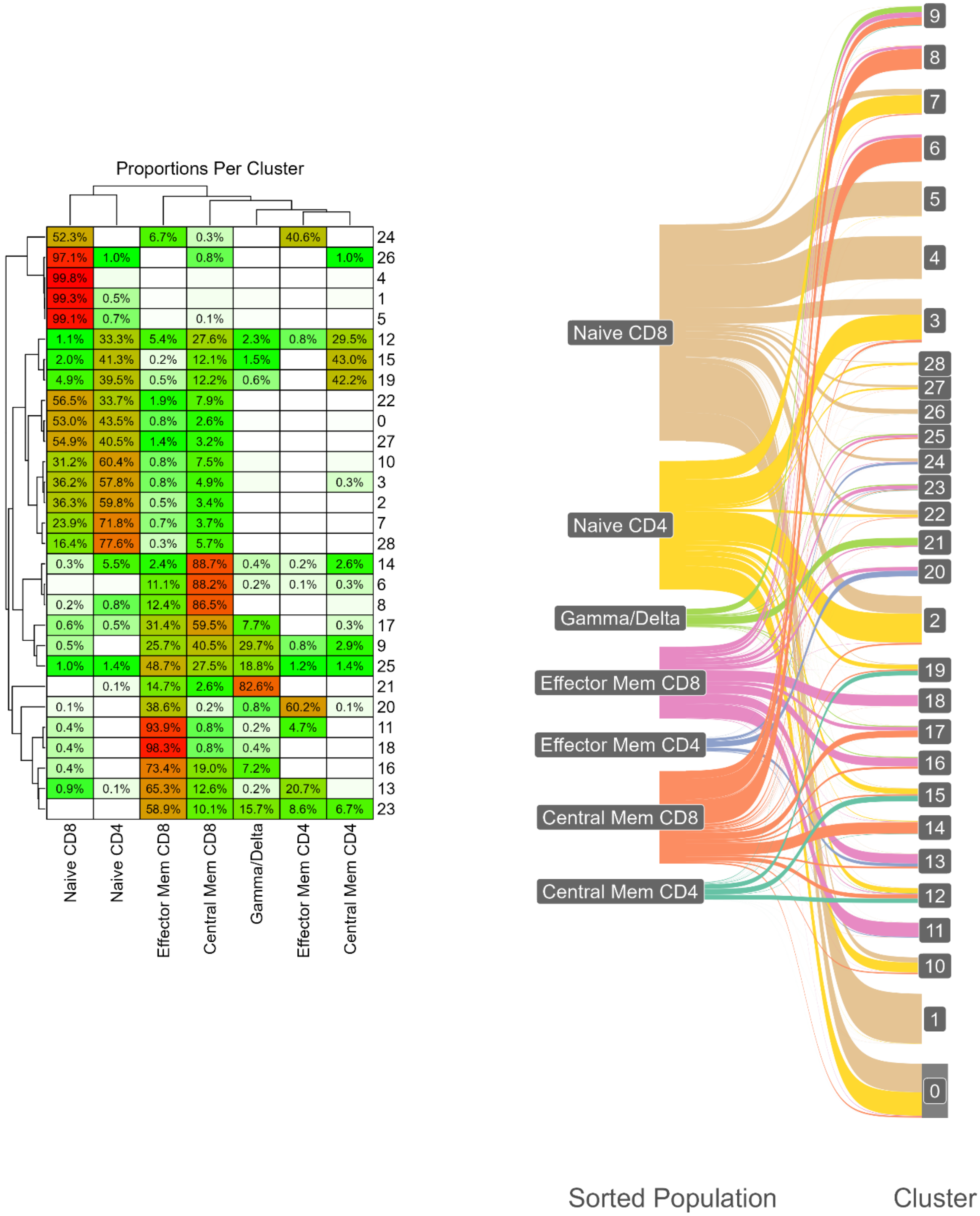

Figures S3. Comparison of unsupervised clustering using different scRNA-seq pipelines and algorithms, related to Figure 5. We performed unsupervised clustering on the data from Figure S1B (ScanPy pipeline) using the louvain clustering algorithm. The heatmap (left) and Sankey diagram (right) contrast the ground-truth population (defined by the cell sort) relative to the cluster assignment. While specific clusters can be generated that are pure or nearly pure for a single population, most clusters are a mixture of reference populations. These data demonstrate that the factors that drive the unsupervised clustering of T cells are not inherently aligned with established lineage definitions.

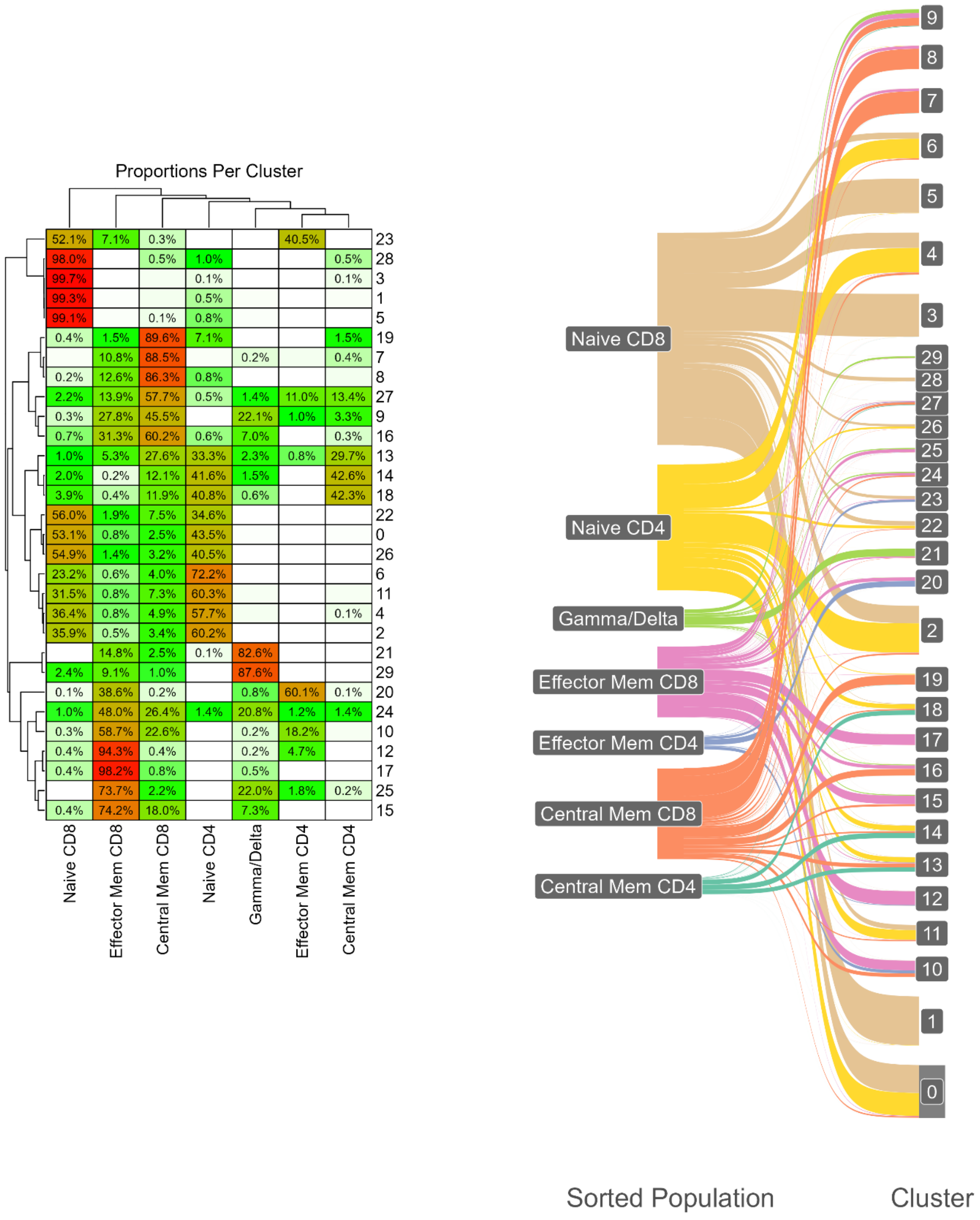

Figures S4. Comparison of unsupervised clustering using different scRNA-seq pipelines and algorithms, related to Figure 5. We performed unsupervised clustering on the data from Figure S1B (ScanPy pipeline) using the louvain-refined clustering algorithm. The heatmap (left) and Sankey diagram (right) contrast the ground-truth population (defined by the cell sort) relative to the cluster assignment. While specific clusters can be generated that are pure or nearly pure for a single population, most clusters are a mixture of reference populations. These data demonstrate that the factors that drive the unsupervised clustering of T cells are not inherently aligned with established lineage definitions.

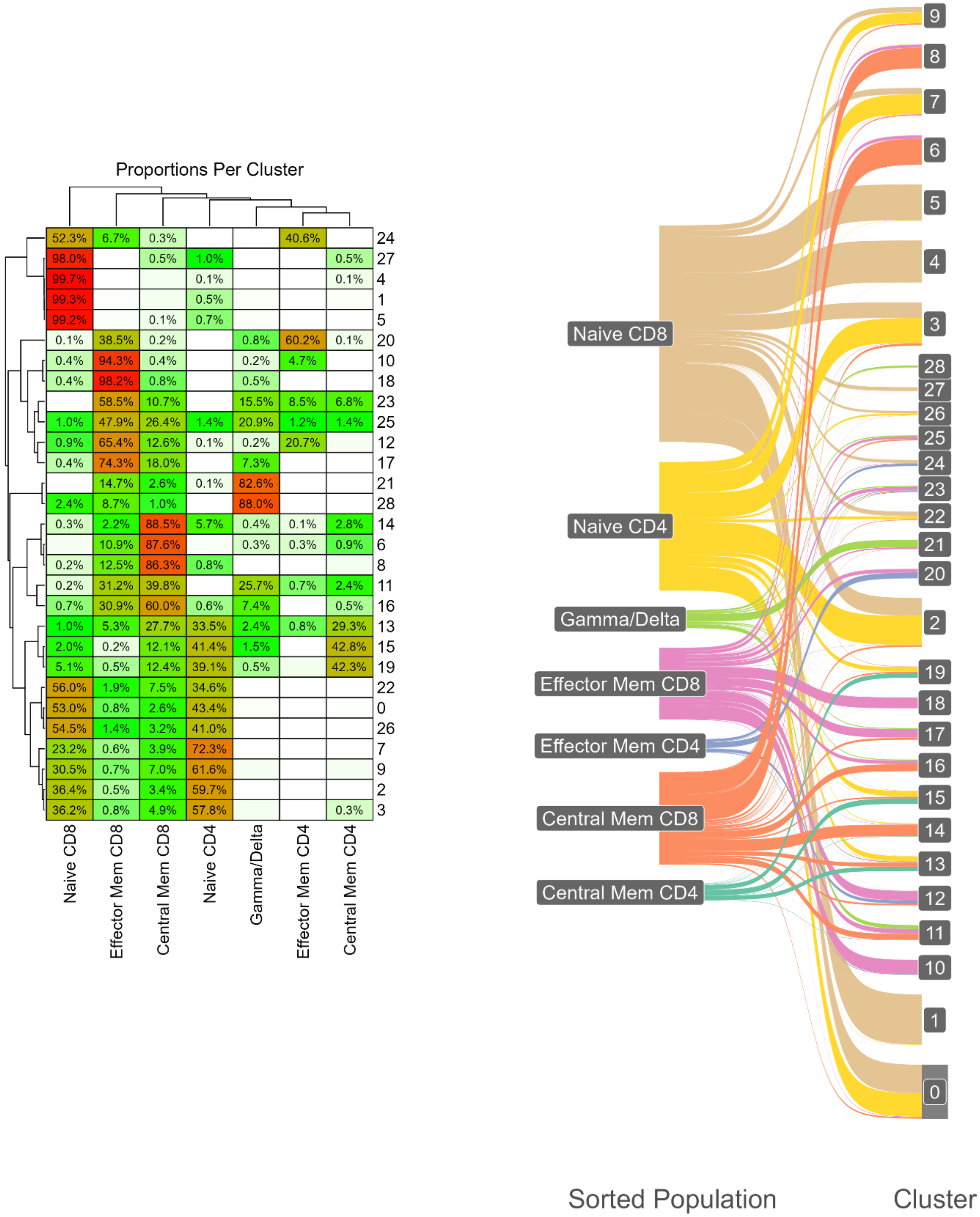

Figures S5. Comparison of unsupervised clustering using different scRNA-seq pipelines and algorithms, related to Figure 5. We performed unsupervised clustering on the data from Figure S1B (ScanPy pipeline) using the SLM clustering algorithm. The heatmap (left) and Sankey diagram (right) contrast the ground-truth population (defined by the cell sort) relative to the cluster assignment. While specific clusters can be generated that are pure or nearly pure for a single population, most clusters are a mixture of reference populations. These data demonstrate that the factors that drive the unsupervised clustering of T cells are not inherently aligned with established lineage definitions.

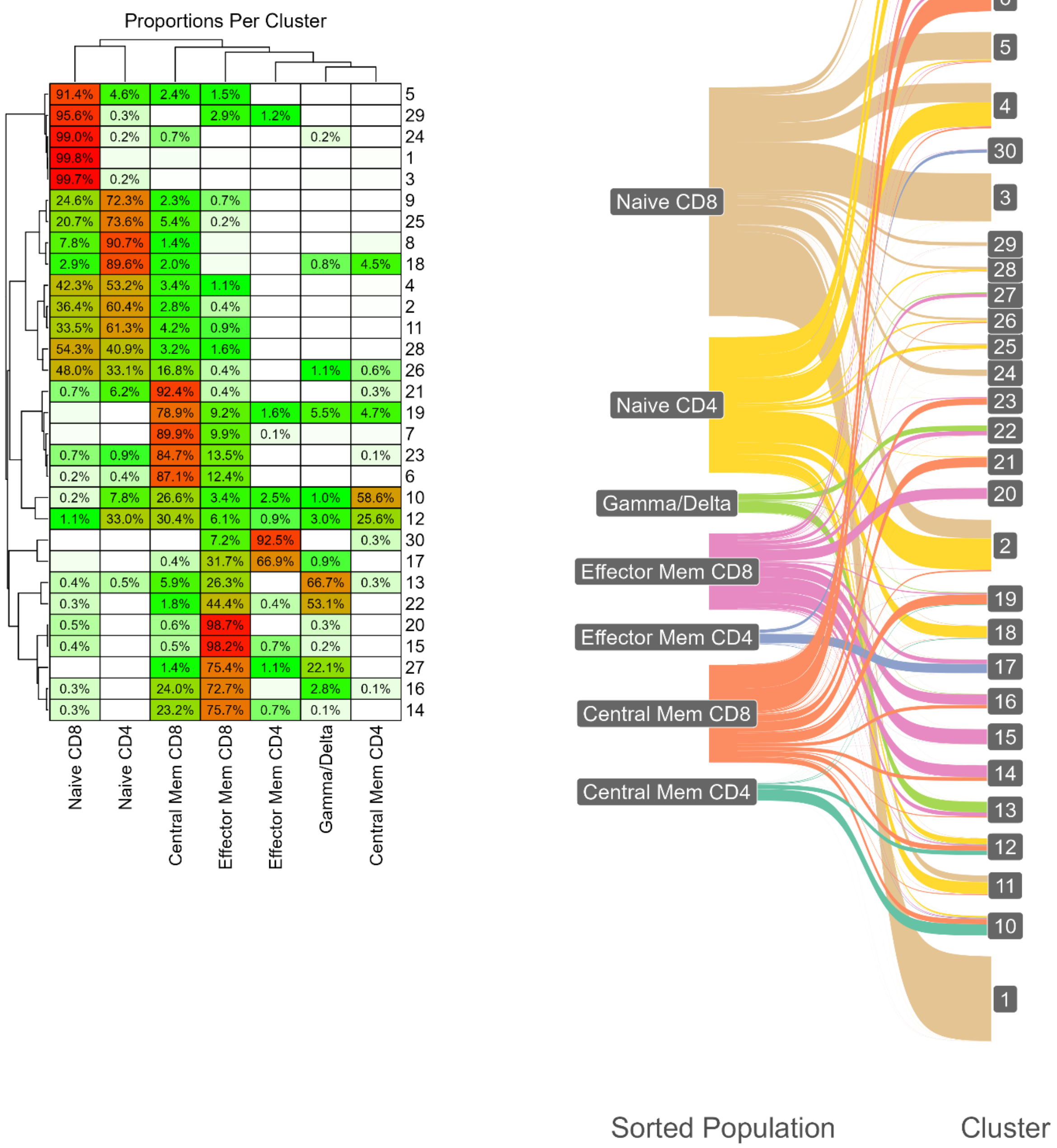

Figures S6. Comparison of unsupervised clustering using different scRNA-seq pipelines and algorithms, related to Figure 5. We performed unsupervised clustering on the data from Figure S1A (Seurat pipeline) using the leiden clustering algorithm. The heatmap (left) and Sankey diagram (right) contrast the ground-truth population (defined by the cell sort) relative to the cluster assignment. While specific clusters can be generated that are pure or nearly pure for a single population, most clusters are a mixture of reference populations. These data demonstrate that the factors that drive the unsupervised clustering of T cells are not inherently aligned with established lineage definitions.

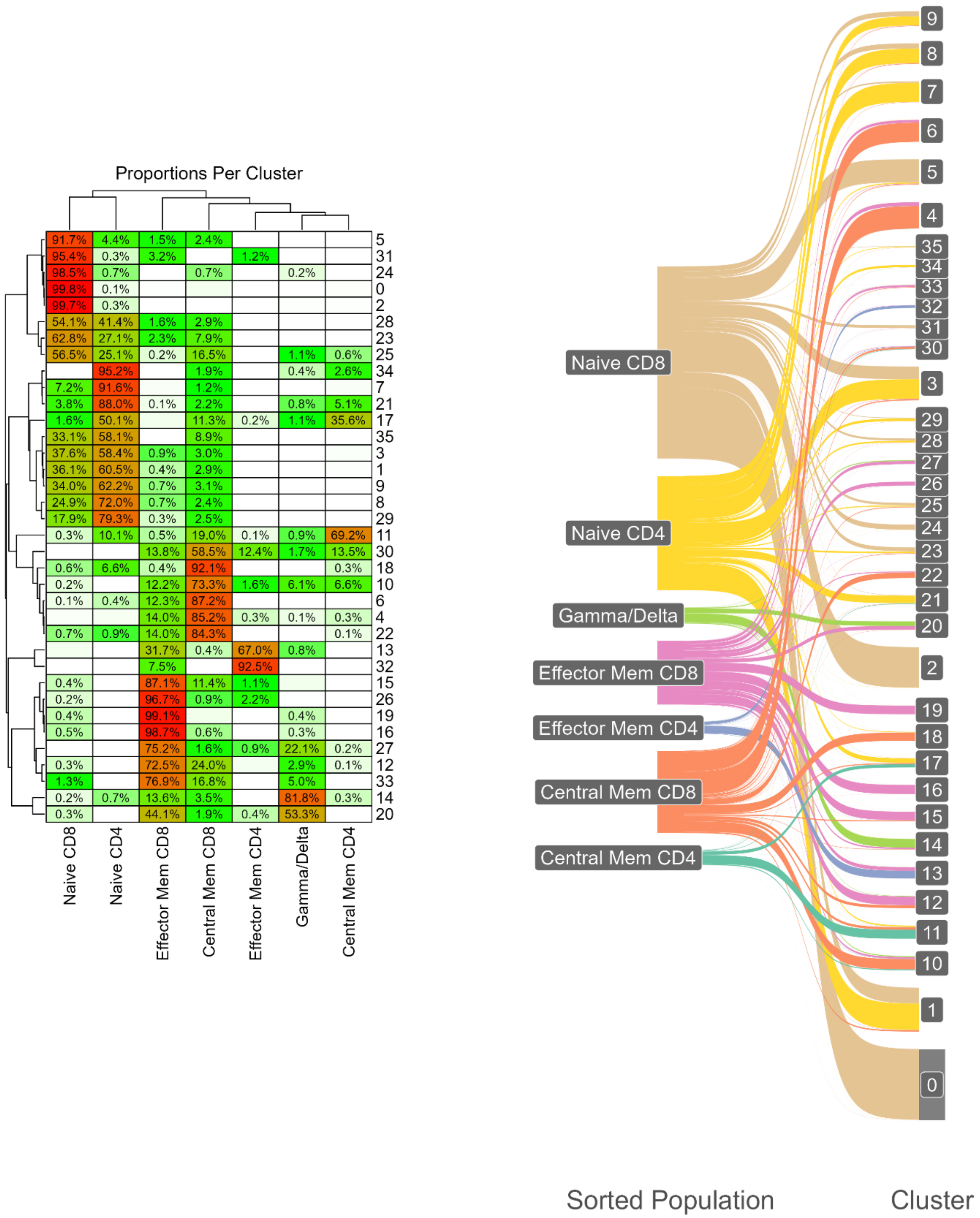

Figures S7. Comparison of unsupervised clustering using different scRNA-seq pipelines and algorithms, related to Figure 5. We performed unsupervised clustering on the data from Figure S1A (Seurat pipeline) using the louvain clustering algorithm. The heatmap (left) and Sankey diagram (right) contrast the ground-truth population (defined by the cell sort) relative to the cluster assignment. While specific clusters can be generated that are pure or nearly pure for a single population, most clusters are a mixture of reference populations. These data demonstrate that the factors that drive the unsupervised clustering of T cells are not inherently aligned with established lineage definitions.

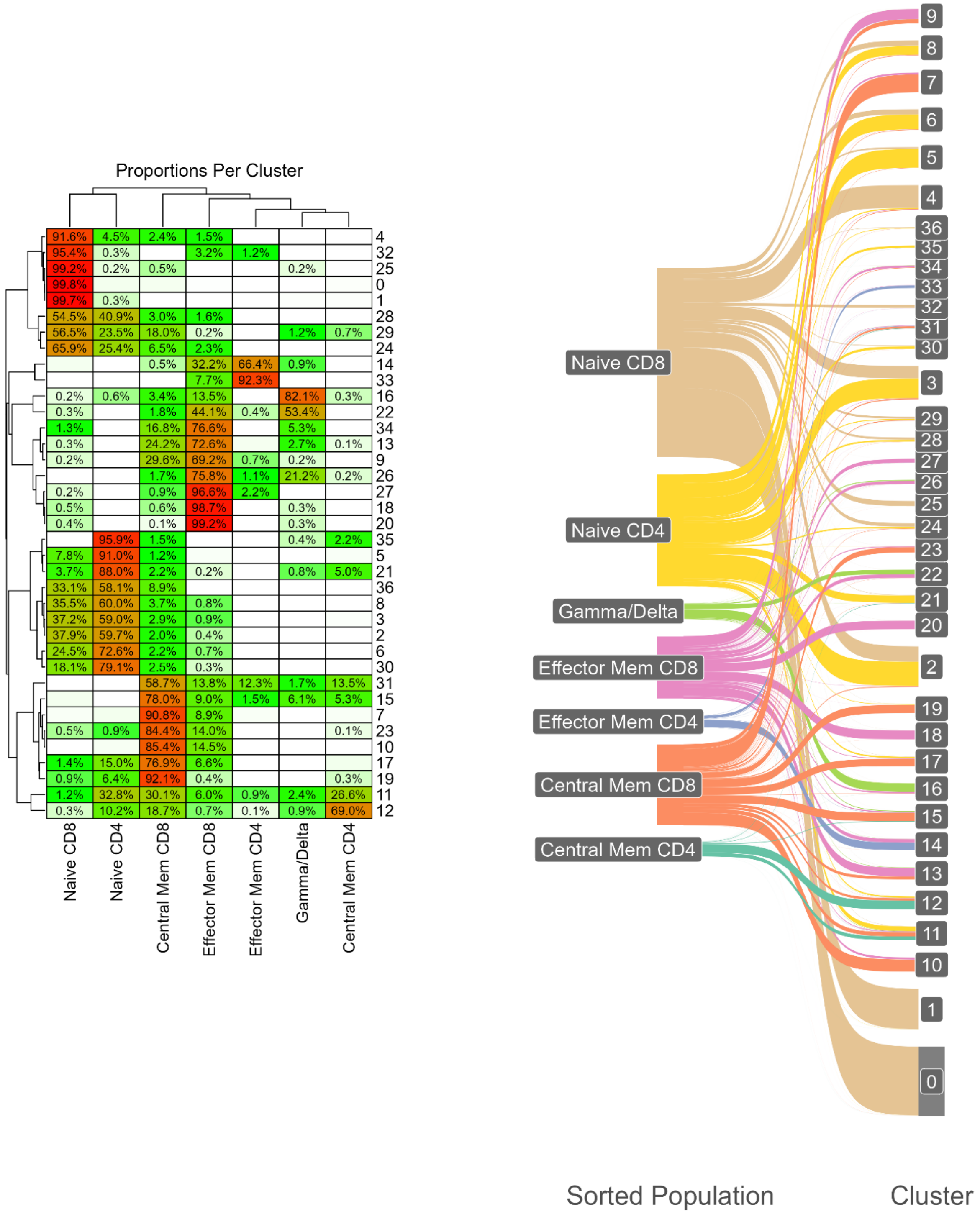

Figures S8. Comparison of unsupervised clustering using different scRNA-seq pipelines and algorithms, related to Figure 5. We performed unsupervised clustering on the data from Figure S1A (Seurat pipeline) using the louvain-refined clustering algorithm. The heatmap (left) and Sankey diagram (right) contrast the ground-truth population (defined by the cell sort) relative to the cluster assignment. While specific clusters can be generated that are pure or nearly pure for a single population, most clusters are a mixture of reference populations. These data demonstrate that the factors that drive the unsupervised clustering of T cells are not inherently aligned with established lineage definitions.

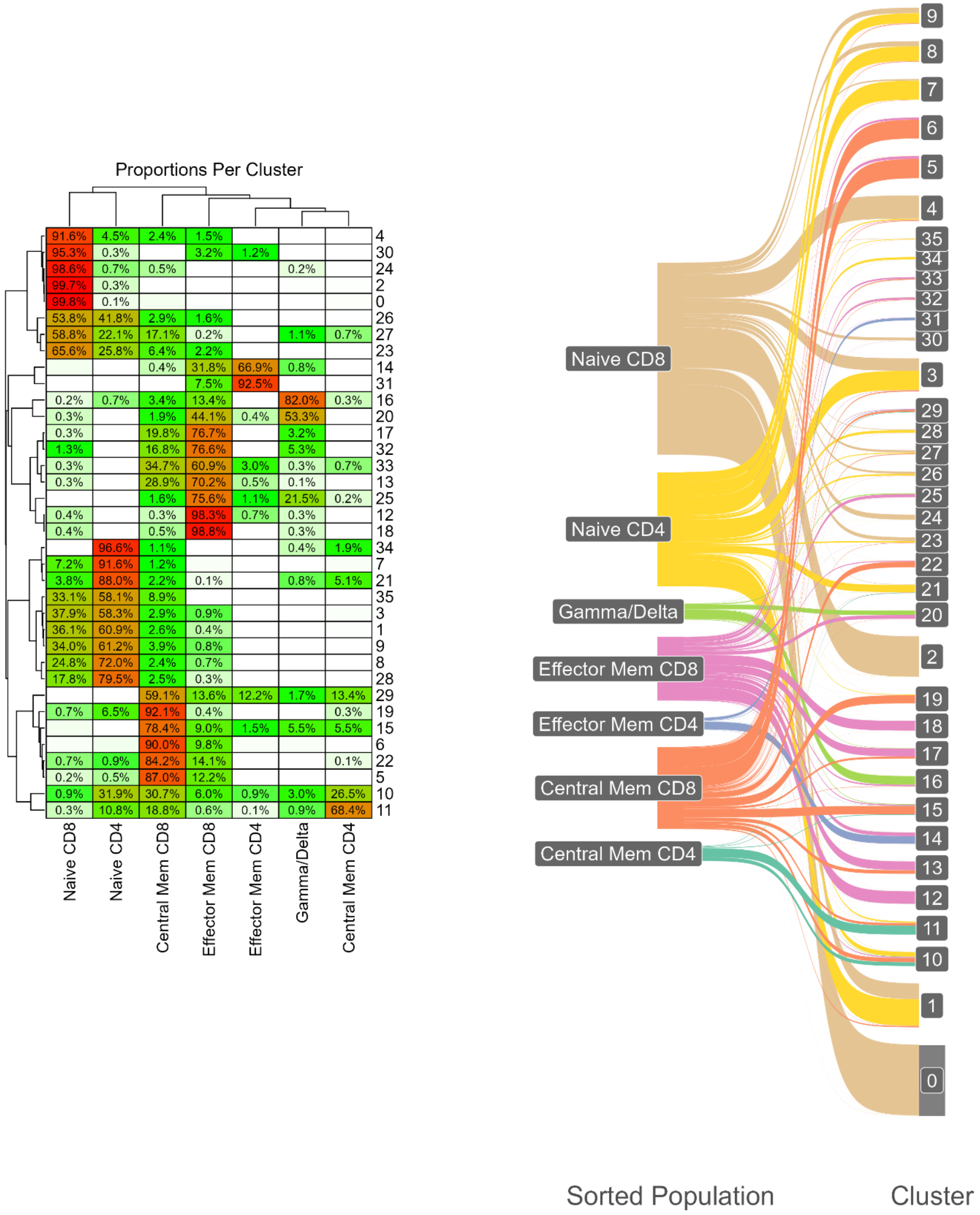

Figures S9. Comparison of unsupervised clustering using different scRNA-seq pipelines and algorithms, related to Figure 5. We performed unsupervised clustering on the data from Figure S1A (Seurat pipeline) using the SLM clustering algorithm. The heatmap (left) and Sankey diagram (right) contrast the ground-truth population (defined by the cell sort) relative to the cluster assignment. While specific clusters can be generated that are pure or nearly pure for a single population, most clusters are a mixture of reference populations. These data demonstrate that the factors that drive the unsupervised clustering of T cells are not inherently aligned with established lineage definitions.

Table S1. Table with demographics information for all rhesus macaques used in this study, related to Figure 1.

Table S2. List of CITE-seq antibodies and concentrations, related to STAR Methods.

Table S3. List of UCell gene modules used for cell type and state scoring, related to Figure 1.

Table S4. List of UCell gene modules used to detect mutually exclusive cell types, related to Figure 1.

Table S5. List of the genes and weights identified for the T cell Effector Differentiation Score (EDS), related to Figure 5.

Table S6. List of primers used to amplify macaque TCR alpha, beta, gamma, and delta sequences, related to STAR Methods.

Table S7. List of genes excluded from top variable genes prior to performing PCA, related to STAR Methods.

Table S8. The reference set of rhesus macaque V/D/J segments used for TCR detection, related to STAR Methods.

Table S9. List of all raw sequence datasets and NIH SRA accession numbers, related to STAR Methods.
